# Predicting overall survival benefit in previously untreated, unresectable or metastatic melanoma from improvement in progression-free survival: a correlation meta-analysis

**DOI:** 10.3389/fonc.2025.1541086

**Published:** 2025-06-05

**Authors:** Peter Mohr, Murat Kurt, Swetha Srinivasan, Andriy Moshyk, Flavia Ejzykowicz, Paul Serafini, Mir-Masoud Pourrahmat, Lisa Leung

**Affiliations:** ^1^ Department of Dermatology, Elbe Kliniken Buxtehude, Buxtehude, Germany; ^2^ Bristol Myers Squibb, Princeton, NJ, United States; ^3^ Evidinno Research Outcomes Inc., Vancouver, BC, Canada

**Keywords:** surrogacy, melanoma, overall survival, progression-free survival, systematic review, meta-analysis

## Abstract

**Objectives:**

To evaluate the association between the treatment effects on progression-free survival (PFS) and overall survival (OS) for previously untreated, unresectable or metastatic melanoma.

**Methods:**

A systematic literature review identified eligible trials reporting PFS and OS. Bivariate random effects meta-analysis (BRMA) was performed to estimate the correlation between the hazard ratios (HRs) of OS (HR_OS_) and PFS (HR_PFS_), and sample size-weighted linear regression (WLR) was used to estimate a surrogacy equation which predict the HR_OS_ from the HR_PFS_. Strength of the correlation obtained from BRMA and WLR models was assessed using published guidelines. Predictive performance of the WLR model was also evaluated internally by leave-one-out cross-validation (LOOCV) and externally against data from newly published trials. Further analyses included adjustments for BRAF mutation status, and restriction to phase III trials or trials evaluating immune checkpoint or BRAF/MEK inhibitors, without crossover or crossover-adjusted, or meeting proportional hazards assumption.

**Results:**

BRMA and WLR estimated a correlation of 0.74 (95%CI: 0.51-0.87) and 0.81 (95%CI: 0.58-0.92), respectively. The estimated surrogacy equation derived from the WLR was lnHR_OS_ = -0.05 + 0.50 × lnHR_PFS_ with a statistically non-significant intercept (95% CI: -0.14 - 0.03) and a statistically significant slope (95% CI: 0.35 - 0.65). The surrogacy equation derived from the BRMA was lnHR_OS_ = -0.11 + 0.36 × lnHR_PFS_ with a statistically non-significant intercept (95% CI: -0.23 - 0.00) and a statistically significant slope (95% CI: 0.17 - 0.57). The predictive accuracy of the WLR was 95.8% in LOOCV. Across sensitivity analyses correlations between HR_PFS_ and HR_OS_ were ≥0.77 and ≥0.85 based on BRMA and WLR, respectively, and the accuracy of the WLR model in LOOCV was ≥88%. When predicting HR_OS_ for newly published trials, the differences between the observed and model-predicted HR_OS_’s were <0.05.

**Conclusions:**

Results suggest a clinically meaningful and moderate trial-level correlation between PFS and OS across all analyses. The analyses and high accuracy of the surrogacy equations shown in internal and external validations can enable earlier prediction of treatment effects on OS from the improvements on PFS for previously untreated unresectable or metastatic melanoma.

## Introduction

Skin cancers are one of the most commonly diagnosed cancers worldwide with melanoma accounting for the majority of skin cancer-related deaths (1). Global estimates from 2020 showed approximately 325,000 new cases of melanoma and 57,000 deaths, which are expected to continue increasing into 2040 (1, 2). Melanoma can be effectively treated if caught early, but in melanomas that were detected after metastasis, the historic 5-year survival rate was low (2) until the development of targeted and immune-oncology therapies that revolutionized the treatment of the disease (3, 4). Median overall survival (OS) in unresectable metastatic melanoma was nearly 6–9 months prior to the introduction of immunotherapies, and can be now as long as 6 years with dual immunotherapy agents (5). The standard of care treatments approved by the US Food and Drug Administration (FDA) for first-line (1L) treatment of melanoma include anti-programmed cell death protein 1 (PD-1) monotherapies, the combination of anti-PD-1 and anti-CTLA-4 therapy, and more recently the combination of anti-PD-1 and anti-LAG-3 therapy as well as BRAF/MEK inhibitors for patients with BRAF mutation (BRAF-MT) (6–8)

OS is the gold standard measure for the evaluation of oncology trials due to its objectivity, patient-centricity and its clinical meaningfulness (9), but demonstrating OS benefit in a randomized setting can require considerable follow-up time especially in settings where there are effective standard of care treatment options. Moreover, OS benefit of a front-line therapy may be confounded by the use of subsequent therapies, availability of which may exhibit differences across local settings. One way to circumvent both issues in the drug development process is to use an appropriately validated surrogate endpoint with an expected shorter time to maturity. Surrogate endpoints can expedite patients’ access to novel, life-extending therapies by reducing the time for development and approval while providing statistical advantages around power, enrollment, and sample size for RCTs (10). As a consequence of this, the use of surrogate endpoints can lead to substantial cost-savings for manufacturers during the design and conduct of clinical trials. In a broader context, they can also contribute to efficient resource allocation and cost-savings at the societal level with their potential to guide physicians in treatment selection and to reduce adverse events, comorbidities and deaths that could otherwise occur in delays during reimbursement evaluation.

Criteria for validating a surrogate endpoint were first proposed by Prentice (11), and since then major regulatory authorities as well as health technology assessment agencies (HTA) have considered biologics license and reimbursement applications based on surrogate endpoints (12, 13). In fact, almost half of the submissions to the FDA for marketing approval of medicines was from clinical trials where surrogate endpoints were primary endpoints (14, 15). A 2021 review of HTA reports from eight agencies found that surrogate endpoints have been considered in coverage and reimbursement decisions in a wide range of cancers including melanoma (12).

Progression-free survival (PFS) is a time-to-event outcome defined as time from randomization until progression or death from all causes, whichever occurs first. PFS has a shorter time to maturity compared to OS as it considers, by definition, both clinical progression and death from all causes as events. Therefore, it is one of the most commonly used surrogate endpoints for OS. PFS is often not impacted subsequent therapies as their initiation may require prior progression event by trial protocol. Therefore, it confines the treatment effect to the current line of therapy. PFS is also included on the FDA’s list of surrogate endpoints that were the basis for drug approval or licensure in melanoma and other solid cancers (16).

PFS has been previously studied as a surrogate endpoint for OS in advanced melanoma. Studies by Flaherty et al. (17), Nie et al. (18), Larkin et al. (19), Mohr et al. (20), and Leung et al. (21) explored different aspects of the surrogacy relationship between PFS and OS in advanced melanoma using different data sources (17–21). Among these, Flaherty et al. (17) used only dacarbazine-controlled RCTs over various lines of therapies in a relatively outdated treatment landscape to estimate the association between the treatment effects on PFS and OS. In other studies, the evidence base was restricted to trials investigating immune checkpoint inhibitor (ICI) therapies in Nie et al. (18) and to only four CheckMate trials in Larkin et al. (19). Both Mohr et al. (20) and Leung et al. (21) investigated multiple surrogate endpoints including PFS using real-world databases, with the former solely analyzing individual-level correlations. As none of these studies used data from recent randomized settings, majority of which investigated ICIs and BRAF/MEK inhibitors, they may not be able to address the impact of recent evolutions in the treatment of metastatic melanoma on the association between the treatment effects on PFS and OS. To fill this major gap in the literature, a correlation meta-analysis was conducted to explore the correlation between the treatment effects on PFS and OS using aggregate level data published from a broad set of RCTs. Sensitivity analyses were performed using different subsets of RCTs to identify the key drivers of the association between PFS and OS.

## Methods

### Systematic literature review

MEDLINE^®^, Embase, and CENTRAL databases were searched up to October 2020, using predefined search strategies. The searches were limited to studies in English and no publication date limits were applied. Keywords included melanoma, immunotherapy, targeted therapy, and chemotherapy, and terms for RCTs. Grey literature searches included conference proceedings between 2018–2020 from the American Society of Clinical Oncology (ASCO), European Society for Medical Oncology (ESMO), Society for Immunotherapy of Cancer (SITC), Society of Melanoma Research (SMR), and the American Association for Cancer Research (AACR).

Eligible RCTs enrolled adults (≥18 years of age) with previously untreated, unresectable or metastatic stage III or IV melanoma. To be eligible for the surrogacy analysis studies had to report either HRs and corresponding 95% confidence intervals (CIs), or Kaplan-Meier (KM) curves, for both PFS and OS. If PFS was not reported, time to progression (TTP) was considered as a proxy for PFS. Although the definition of TTP does not include death, treatment effect on TTP should approximate the treatment effect on PFS fairly well assuming the fraction of PFS events corresponding to death is similar across the two arms. Study selection and data extraction were performed by two independent investigators. All KM curves for PFS and OS were digitized using WebPlotDigitizer to calculate the unreported HRs from the trials and to assess the violation of proportional hazards (PH) assumption. Unreported 95% CIs of HRs were approximated using their standard errors derived from the reported *p*-values.

### Processing input data

Reported HRs from the RCTs assume that the hazards across the arms being compared are proportional over time. Therefore, within each trial, PH assumption was tested for both endpoints to assess whether the reported HRs were statistically representative measures of the treatment effects over time. This was done by reconstructing the underlying time-to-event data in each arm of each RCT utilizing the digitized survival data from the KM curves and the corresponding number-at-risk profiles using the Guyot algorithm (22), then testing the PH assumption with the Global Schoenfeld test (23). Since the Schoenfeld test evaluates the null hypothesis of proportionality, an alpha of 0.1 was used to reduce the chance of concluding proportionality due to low power. Additionally, in studies where HRs were not reported for PFS and OS but KM curves were provided, the underlying HRs were calculated from Cox-PH model using the reconstructed time-to-event data and used in the CMA subsequently.

Analyses for all models were conducted on the natural logarithm-transformed HRs of PFS (lnHR_PFS_) and OS (lnHR_OS_), which is a robust and commonly accepted method of linearizing treatment effects and their relationship. In the visual presentation of the surrogacy equation between the treatment effects, the log-transformed HRs were inverse-transformed to their original scales using the exponential function.

When PFS and OS data were reported from a trial with differing follow-up durations, the estimates from the longest follow-up were utilized in analyses. For trials with three or more randomized arms, which could contribute to the analyses with more than one treatment-control contrast, only one treatment-control contrast was inputted into the model to avoid dependency between the inputs from the same studies.

### Surrogacy models

The association between the log-transformed HRs for PFS and OS was assessed using two models. The first model was a modified bivariate random-effects meta-analysis (BRMA) approach which simultaneously conducts meta-analyses on the two variables and estimates the correlation between the two endpoints (24). Unlike the general BRMA model the modified approach does not require an estimate or an assumption for the within-trial correlation, which suits the available aggregate-level trial data that is used for correlation assessments. Details on computing covariate-adjusted BRMA models are presented in **Supplementary File 1**.

The second approach was a weighted linear regression (WLR) model using the sample size of each trial as its weight. The correlation between the two variables was measured using the Pearson’s coefficient from the WLR and its 95% CI was estimated using bootstrapping.

### Strength of association and model validity

The strength of the association estimated from BRMA and WLR was evaluated according to the German Institute for Quality and Efficiency in Health Care (IQWiG) criteria (25). According to IQWiG, the correlation was labeled as strong if the lower bound of the 95% CI of the estimated correlation >0.85, weak if the upper bound of the 95% CI of the estimated correlation <0.7, and moderate otherwise.

The predictive performance of the surrogacy equations obtained from both BRMA and WLR was assessed using both internal and external validation.

First, internal validation was conducted using leave-one-out cross-validation (LOOCV), in which a model was fitted to the data by omitting one trial at a time and the reported HR_OS_ was compared to the 95% prediction interval (PI) of the predicted HR_OS_ for the omitted trial. According to the National Institute for Health and Care Excellence (NICE) (26), a surrogacy model can be deemed as valid if the reported HR_OS_ is captured by the 95% PIs for at least 95% of the contrasts. The rate at which the significance of the reported HR_OS_ coincided with the significance of the predicted HR_OS_ at a default 95% confidence level was also calculated.

Second, external validation was conducted on 1L advanced melanoma trials that were not in the evidence base but published PFS and OS data after the search date. More specifically, reported and predicted HR_OS_ were compared for IMspire170 (27), PIVOT IO 001 (28), and RELATIVITY-047 trials (29). IMspire170 compared cobimetinib + atezolizumab to pembrolizumab in BRAF wild-type (BRAF-WT) melanoma, PIVOT IO 001 compared the IL-2 agonist bempegaldesleukin combined with nivolumab to nivolumab monotherapy, and RELATIVITY-047 compared the LAG-3-blocking antibody relatlimab combined with nivolumab to nivolumab monotherapy.

For practical implementations of the WLR model, its utility was assessed by estimating the surrogate threshold effect (STE) (30). STEs indicate threshold HR_PFS_ for which the upper bound of the 95% PI around the HR_OS_ is equal to 1 for a trial of a given sample size. A HR_PFS_ less than the estimated STE predicts a favorable HR_OS_ for the intervention arm with a 95% PI below 1. The closer the STE is to 1, the smaller the HR_PFS_ benefit necessary to predict an HR_OS_ benefit, and therefore the greater the practical utility of the model. The larger the sample size of the predicted trial, the closer the STE will be to 1. Because trials in the evidence base recruited between 200 to 300 patients per arm, STEs were reported for two-arm trials with 400 and 600 patients in total, which provides a sense of the range of plausible STEs in practice.

### Software

All analyses were conducted using R (v4.1.1) (31). Reconstructed time-to-event data were derived from digitized KM curves using the ‘digitize’ function from the survHE package in R (32). HRs were calculated and the Schoenfeld test was conducted using the ‘coxph’ and ‘cox.zph’ functions from the survival package of the software (33). BRMA was conducted using the ‘riley’ function from the metamisc package (34). WLR was performed using the ‘lm’ function, weighted correlations were calculated using the ‘cov.wt’ function, and predictions were made using the ‘predict’ function.

### Analysis sets

The primary analysis included the entire evidence base. Sensitivity analyses were conducted for the different subsets of studies in the evidence base as summarized below:

- Trials where both arms were ICIs or BRAF/MEKi to investigate the impact of mechanism of action on the correlation.
- Phase III trials to investigate the impact of sample size of the studies on the correlation.
- Trials that either did not allow crossover or have adjusted treatment effect calculations for crossover to investigate the impact of subsequent treatments on the correlation.
- Trials that did not violate the PH assumption to investigate the impact of using HRs as single measures of treatment effects on the correlation.

Additionally, a weighted multivariate linear regression with an additional covariate representing the percentage of BRAF-MT patients in each trial was also conducted. Impact of BRAF-MT status on surrogacy was investigated to assess the generalizability of the results to different BRAF populations. Studies investigating ICI- or BRAF/MEK inhibitors- were analyzed separately as they better reflect the current research and clinical practice in melanoma treatment. Impact of crossover on the surrogacy was also investigated due to availability of effective treatment options in subsequent lines which in turn could compromise the model’s ability to make inferences on the target 1L population.

## Results

### Evidence base from SLR

**Figure 1:**
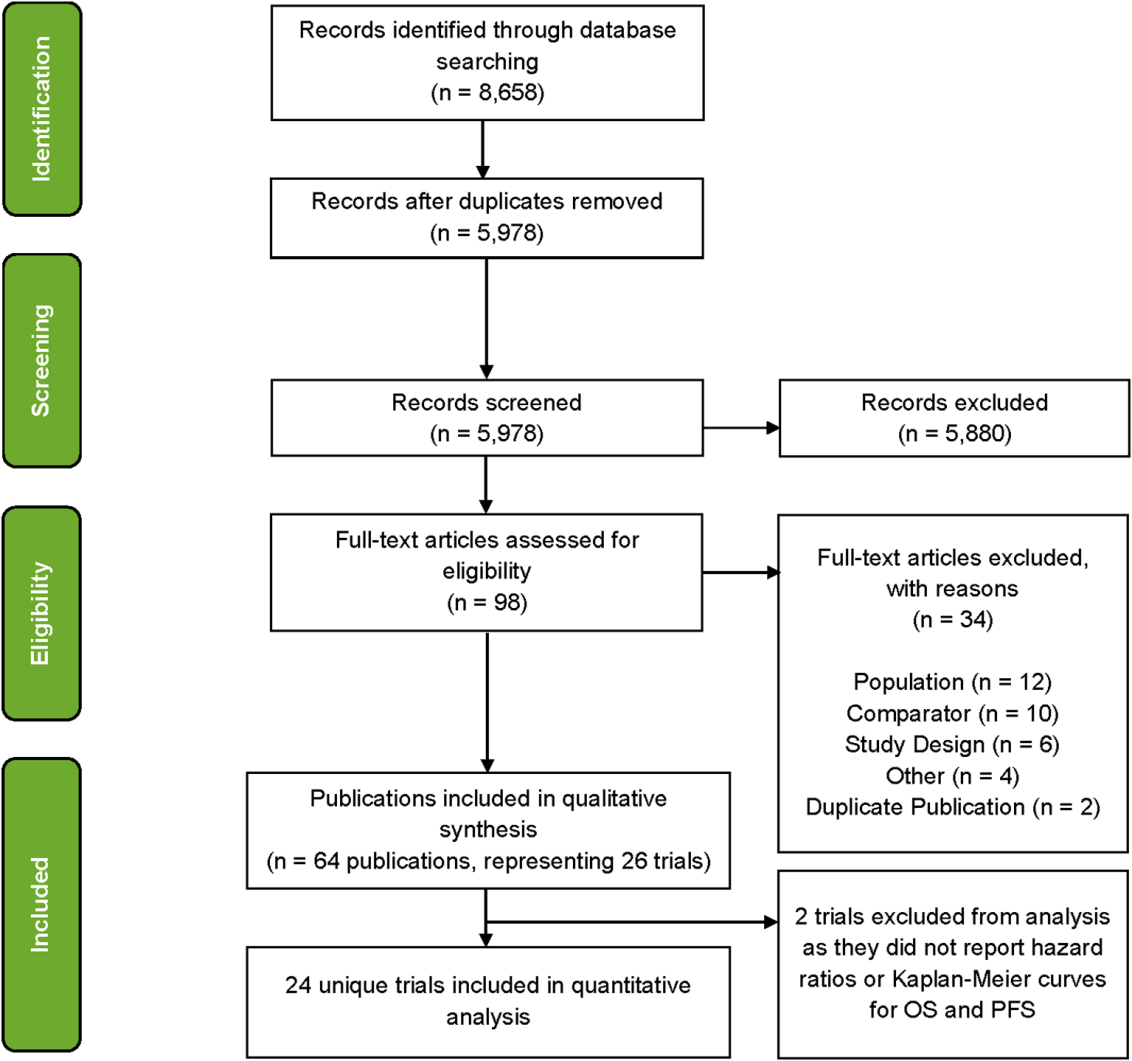
PRISMA flow diagram illustrating the study selection process.

A total of 64 publications associated with 26 trials were identified at the conclusion of the SLR (**Figure 1**) where several trials were represented by multiple publications. After mapping the publications to trials on a one-to-one basis, 38 publications were filtered out and 26 publications were found to be eligible for the evidence base. Of these, for two trials, publications did not report HRs or KM curves for OS and PFS (or TTP as a proxy) and consequently 24 trials were used in analyses (**Table 1**).

**Table 1:** Characteristics of the trials in the evidence base.

Trial	N	Phase	Experimental Arm(s)	Control Arm(s)	BRAF Status (BRAF-MT %)
Algazi (35)	206	II	DBR + TRM (Intermittent)	DBR + TRM (Continuous)	MT (100)
Ascierto (36)	727	III	IPL 10mg	IPL 3mg	Mixed (21.8)
Avril (37)	229	III	FTM	DCR	Not reported
BREAK-3 (38)	250	III	DBR	DCR	MT (100)
BRIM-3 (39)	675	III	VMR	DCR	MT (100)
CheckMate 066 (40)	418	III	NVL	DCR	WT (0)
CheckMate 067 (41)	945	III	NVL + IPL; NVL	IPL	Mixed (31.5)
CheckMate 069 (42)	142	II	NVL + IPL	IPL	Mixed (23.2)
CheckMate 511 (43)	360	IIIb/IV	NVL 3mg + IPL 1mg	NVL 1mg + IPL 3mg	Mixed (41.90)
coBRIM (44)	495	III	VMR + CBM	VMR	MT (100)
COLUMBUS (45)	577	III	ENC + BNM	ENC, VMR	MT (100)
COMBI-d (46)	423	III	DBR + TRM	DBR	MT (100)
COMBI-v (47)	704	III	DBR + TRM	VMR	MT (100)
IMspire150 (48)	514	III	ATZ + VMR + CBM	VMR + CBM	MT (100)
KEYNOTE-006 (49)	834	III	PMB Q2W; PMB Q3W	IPL	Mixed (36.2)
KEYNOTE-022 (50)	120	II	PMB + DBR + TRM	DBR + TRM	MT (100)
KEYNOTE-029 (51)	102	I/II	PMB + IPL 50mg	PMB + IPL 100mg	Mixed (34.3)
Lebbe (52)	194	II	PMS	DCR	Mixed (41.9)
Middleton (53)	305	III	DCR	TMZ	Not reported
NEMO (54)	402	III	BNM	DCR	Not reported
PACMEL (55)	111	II	PCL + PZP; PCL + TRM	PCL	WT (0)
Patel (56)	859	III	TMZ	DCR	Not reported
Robert (57)	502	III	IPL + DCR	DCR	Not reported
Weide (58)	69	IIa	L19IL2 + DCR	DCR	Not reported
IMspire170 (27)	446	III	CBM + ATZ	PMB	WT (0)
PIVOT IO 001 (28)	783	I/II	BEMPEG + NVL	NVL	Mixed (43.2)
RELATIVITY-047 (29)	714	II/III	REL + NVL	NVL	Mixed (38.5)

Shaded rows denote studies that were included in the external validation but not the core analyses or model development.

ATZ, Atezolizumab; BEMPEG, Bempegaldesleukin; BNM, Binimetinib; CBM, Cobimetinib; DBR, Dabrafenib; DCR, Dacarbazine; ENC, Encorafenib; FTM, Fotemustine; IPL, Ipilimumab; MT, Mutant; N, Sample size; NVL, Nivolumab; PCL, Paclitaxel; PZP, Pazopanib; PMB, Pembrolizumab; PMS, Pimasertib; Q2W, Every two weeks; Q3W, Every three weeks; REL, Relatlimab; TMZ, Temozolomide; TRM, Trametinib; VMR, Vemurafenib; WT, Wild-Type.

Across the studies included in the evidence base, median baseline age ranged from 52.2 (38) to 65.0 (40) years (median: 58.6 years), the proportion of male patients ranged from 50.8% (58) to 69.6% (59) (median: 58.7%), and the proportion of White patients ranged from 91.9% (55) to 100.0% (38) (median: 97.6%). The proportion of stage IV patients ranged from 86.6% (42) to 100.0% (37, 38, 53, 56, 58) (median: 95.5%), the proportion of metastatic stage M1c patients ranged from 38.8% (58) to 72.5% (58) (median: 61.0%), and the proportion of patients with brain metastases ranged from 0% (37, 53, 55, 59) to 18.8% (37) (median: 2.5%). The median proportion of patients with ECOG scores of 0 and 1 were 70.7% and 29.1%, respectively. The proportion of patients with a lactate dehydrogenase level above the upper limit of normal ranged from 14.9% (58) to 57.9% (39) (median: 40.0%). Eighteen trials evaluated progression according to RECIST v1.1, four used WHO criteria (36, 37, 53, 57), and one used RECIST v1.0 (56). Median follow-up ranged from 1.7 (54) to 57.7 (46) months with a median of 18.6 months.

In the evidence base, only one trial [Avril et al. (37)] reported TTP but not PFS (37). The exception of using TTP in place of PFS from this trial was the absence of all cause death in the definition of this endpoint unlike PFS. The standard errors of the log-transformed HRs from one trial were calculated from the reported p-values in the absence of CIs (58), and for six trials HRs and their 95% CIs were estimated from reconstructed time-to-event data (37, 38, 46, 55, 59). Five studies in the evidence base had more than two arms: CheckMate 067 (41), PACMEL (55), COLUMBUS (45), KEYNOTE-006 (49), and Weide et al. (58). As both KEYNOTE-006 and Weide et al. (58) studies also reported efficacy results from the data pooling their experimental arms, there was no need to choose a single treatment or comparator arm for the contrast in these trials.

### Primary analysis

The results across all analyses are summarized in **Table 2**.

**Table 2:** Summary of analyses.

Analysis set	Number of included studies	Correlation from the BRMA (95% CI)	Correlation from the WLR (95% CI)	Prediction rate in LOOCV – (OS HR)^†^	Alignment rate in LOOCV – (Significance of OS HR)^††^	STE* (N = 400 – N = 600)
Primary analysis	24 (35–42, 44–48, 52–59)	0.74 (0.51 - 0.87)	0.81 (0.58 - 0.92)	23 (96%)	20 (83%)	0.61 - 0.69
Sensitivity analyses						
BRAF-MT % covariate analysis	18 (35, 36, 38–42, 44–48, 52, 55, 59)	0.77 (0.52 - 0.90)	0.86 (0.60 - 0.95)	18 (100%)	18 (82%)	BRAF-MT:* 0.65 - 0.72 BRAF-WT:* 0.65 - 0.69
ICI- or BRAF/MEKi-only trials	13 (35, 36, 41, 42, 44–48, 59)	0.77 (0.44 - 0.91)	0.88 (0.59 - 0.97)	12 (92%)	11 (85%)	0.74 - 0.80
Phase III trials	17 (36–41, 44–48, 53, 54, 56, 57)	0.86 (0.65 - 0.95)	0.86 (0.62 - 0.95)	15 (88%)	14 (82%)	0.60 - 0.67
Crossover-adjusted	8 (35, 36, 38, 39, 42, 44, 47, 48)	0.92 (0.49 - 0.99)	0.85 (0.32 - 0.98)	8 (100%)	6 (75%)	0.74 - 0.81
PH analysis	8 (35, 37, 41, 42, 47, 53, 56, 59)	0.77 (0.21 - 0.95)	0.86 (0.20 - 0.98)	8 (100%)	7 (88%)	0.51 - 0.58

* – STEs on BRAF-MT was calculated based on BRAF-MT covariate at 100% and BRAF-WT was calculated based on BRAF-MT covariate at 0%, ^†^ – the number and percentage of studies that were accurately predicted in LOOCV, ^††^ – The number and percentage of studies for which the significance of the reported HR_OS_ coincided with the significance of the predicted HR_OS_, BRAF/MEKi, BRAF or mitogen-activated protein kinase inhibitors; Crossover-adjusted, Trials that prohibited or have provided statistically adjusted HR estimates accounting for crossover; ICI, Immune Checkpoint Inhibitor; LOOCV, Leave-One-Out Cross-Validation; N, Sample size; PH, Proportional Hazards; STE, Surrogate Threshold Effect (calculated for a trial with N = 400 and N = 600 patients).

**Figure 2:**
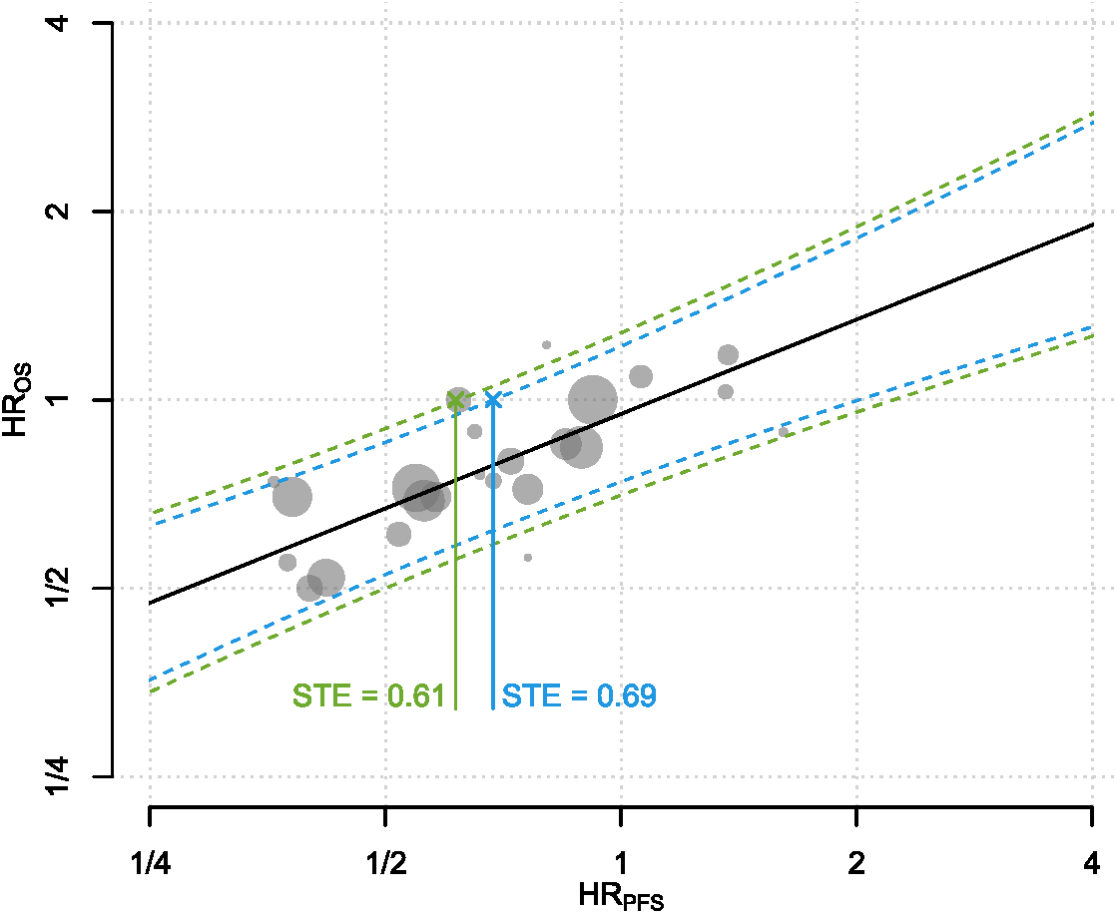
The predictive surrogacy equation is graphed as the solid straight line in black. Each of the plotted gray circles represent the (HR_PFS_, HR_OS_) pair from a treatment-control contrast per trial. Sizes of the circles are proportional to the total number of patients within each contrast. The dotted curves refer to the 95% PIs for the HR_OS_ for a range of HR_RFS_ for hypothetical trials with sample sizes 400 and 600. Solid lines connecting the crosses to the x-axis indicate the STEs calculated for two hypothetical trials with sample sizes 400 (green) and 600 patients (blue). In statistical terms, it corresponds to the HRPFS at which the upper bound of the 95% prediction interval (PI) of the HR_OS_ crosses 1. Both axes are on the logarithmic scale. HR, Hazard Ratio; OS, Overall Survival; PFS, Progression-Free Survival; PI, Prediction Interval; STE, Surrogate Threshold Effect.

In the primary analysis of all 24 studies, BRMA estimated a correlation of 0.74 (95% CI: 0.51 - 0.87) and WLR estimated a correlation of 0.81 (95% CI: 0.58 - 0.92). The estimated surrogacy equation from WLR was lnHR_OS_ = -0.05 + 0.50 × lnHR_PFS_ (**Figure 2**). The intercept of the surrogacy equation derived from WLR was not statistically significant (95% CI: -0.14 - 0.03, *p* = 0.244) however the slope of the equation was statistically significant (95% CI: 0.35 - 0.65, *p* < 0.0001). The estimated surrogacy equation from BRMA was lnHR_OS_ = -0.11 + 0.36 × lnHR_PFS_. The intercept of the surrogacy equation derived from BRMA was not statistically significant (95% CI: -0.23 - 0.00), however the slope of the equation was statistically significant (95% CI: 0.17 - 0.57). The STEs calculated from the WLR for trials with sample sizes of 400 and 600 patients were 0.61 and 0.69, respectively.

**Figure 3:**
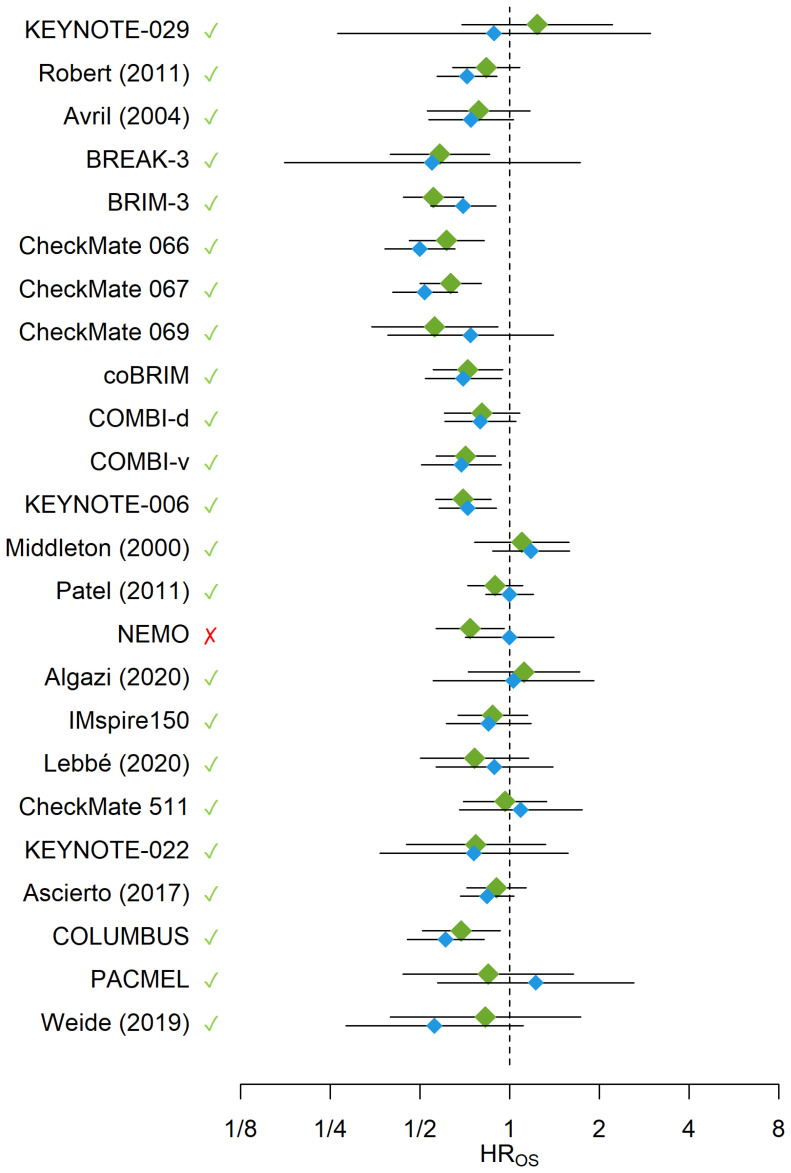
The blue diamonds and their error bars represent the HR_OS_’s and their 95% CIs reported from the trials or calculated from reconstructed survival data, respectively. The green diamonds and their error bars represent the predicted HR_OS_’s and their 95% PIs obtained from the WLR, respectively. The green checkmarks and red crosses indicate whether the observed HR_OS_’s were covered by the 95% PIs generated for the HR_OS_’s from the WLR. The x-axis is on the logarithmic scale. HR, Hazard Ratio; OS, Overall Survival.

In LOOCV (**Figure 3**), the reported HR_OS_ was captured by the 95% PIs of the HR_OS_ for all trials with the exception of NEMO (54) trial, which corresponded to an overall 95.8% accuracy rate for the WLR model. Unlike other trials, the NEMO study enrolled a special group of melanoma patients (NRAS-mutant only) which may potentially explain the outlier behavior of the WLR model for this trial. At a default 95% confidence level for statistical significance, the alignment rate between the significance statuses of the reported and predicted HR_OS’_s was 83.3% (20 out of 24 trials). In 13 trials both reported and predicted HR_OS_’s were not statistically significant whereas in 7 trials both reported and predicted HR_OS_’s were statistically significant. Only in 1 trial, reported HR_OS_ was statistically significant and the predicted HR_OS_ was not statistically significant, and in the remaining 3 trials the observed HR_OS_ was not statistically significant and the predicted HR_OS_ was statistically significant. In 9 out of 24 trials, observed HR_OS_ was greater than the model-predicted HR_OS_ implying over-prediction of OS benefit in the intervention arm by the model. In contrast, in 15 out of 24 trials, observed HR_OS_ was less than the model-predicted HR_OS_ implying under-prediction of OS benefit in the intervention arm by the model. Across the 9 trials where HR_OS_ was under-predicted by the model, the average under-prediction margin was 0.16, whereas across the 15 trials where HR_OS_ was over-predicted by the model, the average over-prediction margin was 0.09.

### Sensitivity analyses

The analysis with BRAF-MT status as a continuous covariate in the WLR included 18 studies. BRMA estimated a correlation of 0.77 (95% CI: 0.52 - 0.90) and WLR estimated a correlation of 0.86 (95% CI: 0.60 - 0.95). The estimated surrogacy equation from the WLR was lnHR_OS_ = -0.04 - 0.09 × BRAF-MT + 0.63 × lnHR_PFS_ - 0.28 × lnHR_PFS_ × BRAF-MT, where the continuous variable “BRAF-MT” represents the fraction of BRAF-MT patients in a study. Results of this covariate-adjusted analysis for a population entirely consisting of BRAF-MT patients is plotted in **Supplementary Figure S1** and the results for a population with no BRAF-MT patients is plotted **Supplementary Figure S2**. The estimated STEs for trials with 400 and 600 patients were 0.65 and 0.72, respectively, for a trial consisting of BRAF-MT patients entirely, whereas for a trial with no BRAF-MT patients the STEs corresponding to 400 and 600 patients were estimated as 0.65 and 0.69, respectively. After adjusting for BRAF-MT status, in LOOCV (**Supplementary Figure S3**), the reported HR_OS_’s were captured by the 95% PIs for the predicted HR_OS_’s generated by the WLR for all trials. In LOOCV, the alignment rate between the significance of the reported and predicted HR_OS_’s was 82% (i.e. for 20 out of 24 trials).

The remaining sensitivity analyses conducted on selected subsets of the studies generated comparably strong results as the primary analysis. A summary of the results from the sensitivity analyses is presented from **Supplementary Figure S4** to **Supplementary Figure S11**. From the BRMA, correlation estimates ranged from 0.77 to 0.92, and from the WLR correlation estimates ranged from 0.86 to 0.88. Across all sensitivity analyses, the coverage rates of the observed HR_OS_’s by the 95% PIs generated from the WLR was ≥88%, and the alignment rate between the significance of the reported and predicted HR_OS_ was ≥80% among all trials. For trials with 400 and 600 patients, respectively, STEs ranged between 0.51 and 0.58 when the analyses were restricted to trials that did not fail the proportionality assumption, between 0.74 and 0.80 when the analyses were restricted to trials investigating ICI- or BRAF/MEKi-only, and between 0.74 and 0.81 when restricted to trials that adjusted for crossover.

For the primary analysis, correlations obtained from the BRMA and WLR indicated moderate strength between the treatment effects of PFS and OS. Sensitivity analyses from both models also indicated moderate correlation between the HR_PFS_’s and HR_OS_’s. Relative to primary analyses and other sets of sensitivity analyses, correlation estimates were stronger when the analyses were restricted to phase III studies and trials reporting treatment effects that are adjusted with crossover. The correlations between the treatment effects on PFS and OS in these two selected subsets of trials were also stronger than their counterparts computed from the BRAF-adjusted model.

### External validation

Primary model predictions for IMspire170, PIVOT IO 001, and RELATIVITY-047 trials generated OS HRs that are close to those reported from the trials (**Table 3**) (27–29).Across the three studies, the largest gap between the reported and model-predicted OS HRs obtained from the secondary model adjusting for BRAF-MT status was 0.03, compared to 0.05 using the primary model. Therefore, overall, predictions from the secondary model using the proportion of BRAF-MT as a continuous covariate were more accurate than the primary model.

**Table 3:** Results of external validation using the primary and secondary models.

					HR_OS_ (predicted)	
Trial	N (Sample Size)	BRAF status	HR_PFS_ (observed)	HR_OS_ (observed)	Primary model	Secondary model
IMspire170 (27)	446	Wildtype (0% MT)	1.15 (95% CI: 0.88 - 1.50)	1.06 (95% CI: 0.69 - 1.61)	1.02 (95% PI: 0.76 - 1.36)	1.05 (95% PI: 0.73 - 1.52)
PIVOT IO 001 (28)	783	Mixed (41.1% MT)	1.09 (95% CI: 0.88 - 1.35)	0.94 (99.93% CI: 0.71 - 1.24)	0.99 (95% PI: 0.79 - 1.24)	0.97 (95% PI: 0.76 - 1.23)
RELATIVITY-047 (29)	714	Mixed (38.5% MT)	0.75 (95% CI: 0.60 - 0.90)	0.80 (95% CI: 0.60 - 1.00)	0.82 (95% PI: 0.66 - 1.03)	0.80 (95% PI: 0.64 - 1.00)

The secondary model adjusts for proportion of BRAF mutants as a continuous covariate.

CI, Confidence Interval; HR, Hazard Ratio; MT, mutant; N, Sample size; OS, Overall Survival; PFS, Progression-Free Survival; PI, Prediction Interval.

## Discussion

PFS was assessed as a surrogate endpoint for OS in 1L melanoma (*de-novo* metastatic disease with no prior exposure to surgery or adjuvant therapy). The correlations between treatment effects of PFS and OS were moderately strong per IQWiG and clinically meaningful in the primary analysis. Following NICE’s guidance on assessing model validity (26), the internal cross-validations indicate PFS as a valid surrogate for OS. This was further supported by the external validation of the model against three studies published after the search date for the SLR. For each of these major Phase III trials, regardless of the inclusion of BRAF-MT status as a covariate in the model, HR_OS_ predictions were close to their reported counterparts. Additionally, the STEs for trials with at least 400 patients were relatively achievable, and hence the surrogacy model has high practical value for clinicians as well as statisticians and practitioners engaged in clinical trial design. BRAF-MT status as a covariate modestly affected the slope of the surrogacy equation, and the STEs were minimally sensitive to the fraction of BRAF-MT patients for a trial with 400 patients. Various sensitivity analyses generated similar or better correlations compared to the primary analysis, and consistently pointed out moderate strength for the association between the treatment effects per IQWiG indicating the robustness of the model and evidence base. Additionally, although all correlations were moderately strong according to IQWiG criteria based on their 95% CIs, point estimates were high and clinically meaningful.

The results of the WLR were similar to those obtained from previous surrogacy analyses in 1L advanced melanoma literature employing WLR. Analyses conducted by Flaherty et al. (17) using 12 dacarbazine-controlled RCTs identified in an SLR (17), Nie et al. (18) using eight RCTs identified in an SLR of anti-PD-1 and anti-programmed death-ligand 1 therapies (18), and Larkin et al. (19) using four ICI trials have all utilized WLR in exploring the trial-level association between PFS and OS (19). Flaherty et al. (17) reported a correlation of 0.89 (95% CI: 0.68 - 0.97), which is slightly larger than the finding of the WLR in this study (0.81), possible due to the inclusion of more recent trials in the present study; the options for second line treatment have improved since the Flaherty et al. analysis was conducted, and this might have impacted the association. In the subgroup analysis of 1L trials by Nie et al. (18), the estimated R^2^ of 0.91 (95% CI: 0.51 - 0.99) was higher than this study’s (0.65; 95% CI: 0.34 - 0.82). On the other hand, the R^2^ estimate from Larkin et al. (19) was only slightly higher than the one from this study (0.71), albeit with a wider CI than (95% CI: 0.23 - 1.00), likely due to the smaller number of studies included in that surrogacy analysis.

Development of modern immunotherapy agents, targeted therapies and antibody-drug conjugates has transformed the treatment of several advanced stage cancers including melanoma which were historically associated with poor prognosis (60–65). As collection of statistically mature OS data may require several years in these cancers, linking disease progression to death in a statistical and causal pathway gained further clinical importance not only for the timely selection of most appropriate therapies for patients but also for more efficient trial designs. As strength of correlation between PFS and OS depends on several factors including disease stage and physiology, subsequent treatment patterns, mechanisms of action of the investigated therapy class, and biomarkers, surrogate endpoint validation is a demanding procedure that must be undertaken individually for each clinical context. By its systematic approach from the generation of evidence base to the design of primary and sensitivity analyses with respect to key disease-specific determinants of the correlation and exploration of two separate methodologies to measure the robustness of the outcomes with respect to parameterization and choice of model, our study provides a blueprint for the exploration of PFS as a surrogate for OS in other cancers.

This surrogacy analysis of two dozen RCTs is the most comprehensive to date in the literature of 1L advanced melanoma. Prior to this study, the largest of the aforementioned analyses was Flaherty et al. (17), which included an evidence base including not only older therapies that are no longer considered standard practice but also therapies that are used in later lines of treatment. Furthermore, the analyses in our study included a wider range of therapies with a subgroup analysis for contemporary ICI and BRAF/MEKi therapies, which will improve the generalizability of our results to a wide range of therapies and to more recent therapies. Other strengths of this analysis are (i) the assessment of the validity of the PH assumption for all studies with a sensitivity analysis excluding those studies that failed it, (ii) an external validation vs. new published trials which showed high accuracy, (iii) the use of BRMA in addition to WLR, which serves as an internal validation mechanism while utilizing different level of input from the evidence than WLR, and (iv) employing a novel extension of BRMA incorporating additional variables to adjust for the BRAF-MT status as a key prognostic factor. Compared to WLR, BRMA has been an endorsed approach by NICE and unlike the WLR it incorporates the standard errors of both endpoints into assessment. Lastly, to aid future research, a standalone R function for predicting OS for the primary model was developed (**Supplementary Figure S12**).

To conclusively validate PFS as a surrogate for OS, it is necessary to demonstrate all three levels of evidence: (1) a treatment-level association between PFS and OS, (2) an individual-level association between PFS and OS, and (3) the biological plausibility of a causal relationship between PFS and OS (66). Notably, the scope of this study was limited to establishing only treatment-level association which indeed is the most critical type of evidence to be utilized in the design and evaluation of new clinical trials. Unlike Larkin et al. (19), this study did not have access to the individual patient data from the trials in the evidence base which would be needed to establish an individual-level association. Demonstration of the biological plausibility of a causal relationship is beyond the scope of a correlation meta-analysis. Nevertheless, with no proven implication, treatment-level association is often consistent with an individual-level association. Therefore, our study suggests the evidence on individual-level association is worth investigating in future studies. Additionally, further validation of PFS in melanoma—through mechanistic, epidemiologic, and clinical data—may help support its broader acceptance as a surrogate endpoint in both clinical and regulatory decision-making.

This study had three minor limitations that should be acknowledged.

First, only eight of the trials in the evidence base performed crossover-adjusted analyses for their efficacy data. As depicted in **Supplementary Figure S13**, although pairs of log-transformed HR_PFS_ and HR_OS_ do not show a visible variation from the general trend of the data and are well aligned around the estimated surrogacy equation, the presence of crossover is shown to dilute the strength of correlation. Crossover is not only a common phenomenon to randomized settings but also reflective of real-world clinical practice, where patients are not subject to clinical trial protocols and may switch between a variety of treatments based on the discretion of their physicians. Therefore, from a practical standpoint, including trials with crossover in the evidence base not only enhances the generalizability of the findings to real-world settings but also enables decision makers to predict the effects of crossover on the estimated OS benefit. However, generalizability of our findings from the primary analysis may be limited to settings where subsequent treatment patterns show similarities to the observed trends across the trials included in our evidence base. Furthermore, due to limited number of studies reporting crossover-adjusted data, under both WLR and BRMA, there was substantial uncertainty around the estimated correlation within these studies. Therefore, generalizability of the insights from the analysis of this subset of studies to broader settings should be approached with caution.

In our study, the absence of patient-level data or more granular aggregate-level information on crossover (i.e. rates and average timings of crossover) from the trials limited the applicability of a more advanced analysis that could investigate the impact of crossover on the strength of correlation. With more aggregate level data from the trials, a promising yet sophisticated future research direction can consider extending both WLR and BRMA to multivariate basis with covariates such as crossover rate, average timing of crossover from randomization, and the difference between the mechanisms of actions of experimental and control arm therapies in each trial. On the other hand, with patient level data from the trials in the evidence base, a more streamlined future research direction can re-calculate OS HRs using advanced methods (e.g. Rank Preserving Structural Failure Time Models Iterative Parameter Estimation algorithm, Inverse Probability of Censoring Weights) adjusting for the rates and timings of crossover before being analyzed with PFS HRs via WLR and BRMA.

Second, in our evidence base, only Avril et al. (37) did not report HR_PFS_ (37). In the absence of this information, HR_TTP_ from this study was used in place of HR_PFS_. Based on the assumption that the frequency of pre-progression death events was similar across treatment arms, HR_TTP_ is expected to approximate the unreported HR_PFS_ in this study. When compared with the input data from other studies in the evidence base, the HR_TTP_ and HR_OS_ reported by Avril et al. (37) were close to the medians of the HR_PFS_ and HR_OS_ data across the rest of the evidence base, respectively, suggesting input data used from Avril et al. (37) do not show any tendency to skew the results.

Third, regardless of the approach, the estimated correlations from the primary analysis did not meet the threshold according to IQWiG to be classified as strong. Unlike IQWiG, criteria by Biomarker-Surrogacy Evaluation Schema 3 (BSES3) (67) consider R² when labeling the strength of the correlation between PFS and OS but it does not take the 95% CI of the correlation coefficient (r) or R² into account. According to BSES3, correlation between PFS and OS can be categorized as “excellent” if R² ≥ 0.6 and as “good” if 0.6 > R² ≥ 0.4. Therefore, in our case, according to BSES3, the correlation obtained from WLR (R² = 0.66) could be categorized as excellent whereas the correlation obtained from BRMA (R² = 0.55) could be categorized as good. Besides the variety across published guidelines in assessing the strength of a correlation, in addition to the estimated correlation coefficient (r) or R², the model’s predictive performance may play a vital role for the acceptability of PFS as a valid surrogate endpoint for OS. Internal cross validation experiments show 95.8% alignment between the observed OS HRs and the 95% PIs for OS HR predicted from PFS HR emphasizing model validity according to NICE criteria (26) and the predictive value of PFS benefit in earlier estimation of OS benefit. Thus, coupled with the variety of criteria across local and published guidelines for surrogate endpoint validation, differences between the estimated correlations from WLR and BRMA approaches may not warrant a uniform view on the acceptance of PFS as a strong predictor of OS in previously untreated metastatic melanoma and require further research on the subject.

While PFS is commonly used as a (co)-primary endpoint in first-line metastatic melanoma trials—appearing in 13 of the 24 studies in our evidence base—and is often considered a valid surrogate for OS in this context, the association between PFS and OS has not been formally assessed using the most recent trials despite transformative advances in immunotherapy and targeted therapies. Our study aims to formalize this association only from a statistical standpoint by deriving various summary measures (e.g. correlation coefficients, 95% CIs around the slope and intercept of surrogacy equations, surrogate threshold effect) that would enable the interpretation of results by practitioners and regulatory agencies. Despite comprehensive analyses and statistical insights derived in our study, acceptance of PFS as a valid surrogate endpoint for OS in previously untreated metastatic melanoma by regulatory agencies depend on the class of treatment and guidelines used to evaluate the strength of the correlation, and require complementary statistical, clinical, epidemiological and biological evidence, generation of which were beyond the scope of our research.

## Conclusions

This study demonstrates PFS as a valid surrogate for OS when defined by NICE criteria, while the strength of the correlation is labeled as moderate according to IQWiG criteria, and good-to-strong according to BSES3 criteria depending on the methodology used to derive the correlation. The estimated range of STEs based on the sample sizes of recent major trials show the practical value of surrogacy equation for rapid clinical insights and the designs of future trials. Overall, the results suggest that HR_PFS_ can be used as a surrogate endpoint for HR_OS_ in the 1L setting for unresectable/metastatic melanoma.

## Data Availability

The original contributions presented in the study are included in the article/**Supplementary Material**. Further inquiries can be directed to the corresponding author.
