# Supplementary material for: Predicting overall survival benefit in previously untreated, unresectable or metastatic melanoma from improvement in progression-free survival: a correlation meta-analysis
Source: Front Oncol. 2025 Jun 5;15:1541086. doi: 10.3389/fonc.2025.1541086 (PMC12176743; doi:10.3389/fonc.2025.1541086)
Supplement: Supplementary file 1 [file SupplementaryFile1.docx]

**Supplementary Materials**

**Supplementary File 1:** Details of computing correlation estimates on models with more than one predictor.

This section summarizes the adjustments made to the WLR and BRMA procedures to handle the addition of a variable representing the fraction of BRAF-MT patients in each study.

In WLR, the relationship between lnHR_OS_ and lnHR_PFS_ combined with the proportion of BRAF-MT patients was modelled using weighted multiple linear regression. The strength of association between lnHR_OS_ and the two predictors (lnHR_PFS_ and the proportion of BRAF-MT) was estimated using the coefficient of multiple correlation: the correlation between the observed and predicted values of lnHR_OS_ for the studies in the evidence base. This is a value ranging from 0 (no association) to 1 (perfect correlation).

Since BRMA only supports two input variables of the true endpoint and the surrogate endpoint, it was necessary to reduce the two predictors (lnHR_PFS_ and BRAF-MT fraction) to a single surrogate variable. We did this by using the predicted lnHR_OS_ from the WLR model as the surrogate variable after transforming it to have the same mean and standard deviation as lnHR_PFS_ by applying

$$mean(y) + (x - mean(x)) \times(SD(y) / SD(x)),$$

where *y* is a vector of observed lnHR_OS_ values from the studies, and *x* is a vector of lnHR_OS_ predictions for the studies from the WLR model. This procedure is analogous to the method for calculating the coefficient of multiple correlation in WLR.

**Figure S1**: Weighted linear regression model adjusting for proportion of BRAF-MT patients (100% BRAF-MT).


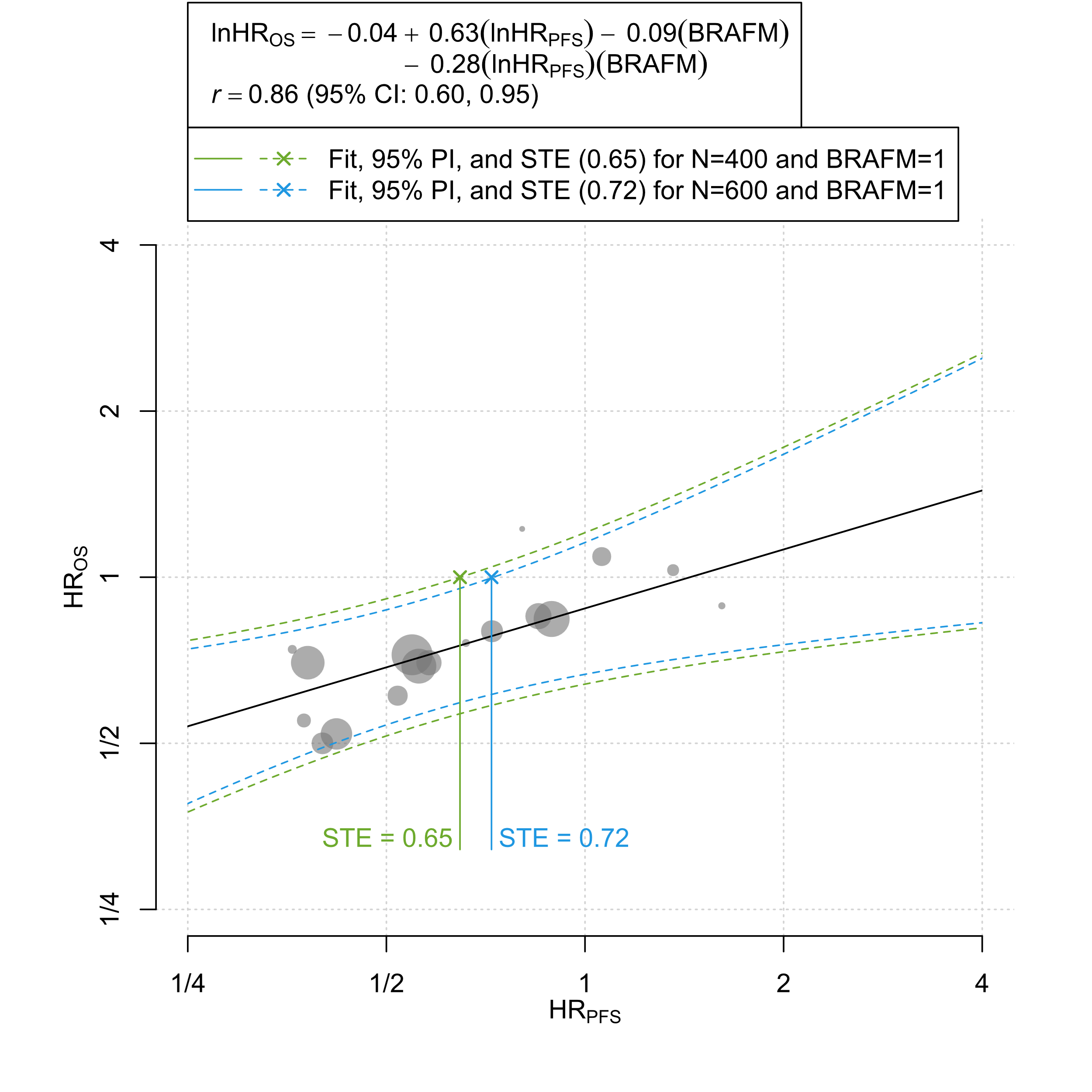


The predictive surrogacy equation (the solid straight line in black) from the multivariate WLR adjusting for the proportion of BRAF-MT patients. The demonstration is for the populations consisting of entirely BRAF-MT patients. Each of the plotted gray circles represent the (HR_PFS_, HR_OS_) pair from a treatment-control contrast per trial. Sizes of the circles are proportional to the total number of patients within each contrast. The dotted curves refer to the 95% PIs for the HR_OS_ for a range of HR_RFS_ for hypothetical trials with sample sizes 400 and 600. Solid lines connecting the crosses to the x-axis indicate the STEs calculated for two hypothetical trials with sample sizes 400 (green) and 600 patients (blue). In statistical terms, it corresponds to the HR_PFS_ at which the upper bound of the 95% PI of the HR_OS_ crosses 1. Both axes are on the logarithmic scale.

Abbreviations: CI – Confidence Interval, HR – Hazard Ratio, OS – Overall Survival, PFS – Progression-Free Survival, PI – Prediction Interval, STE – Surrogate Threshold Effect.

**Figure S2**: Weighted linear regression model adjusting for proportion of BRAF-MT patients (0% BRAF-MT).


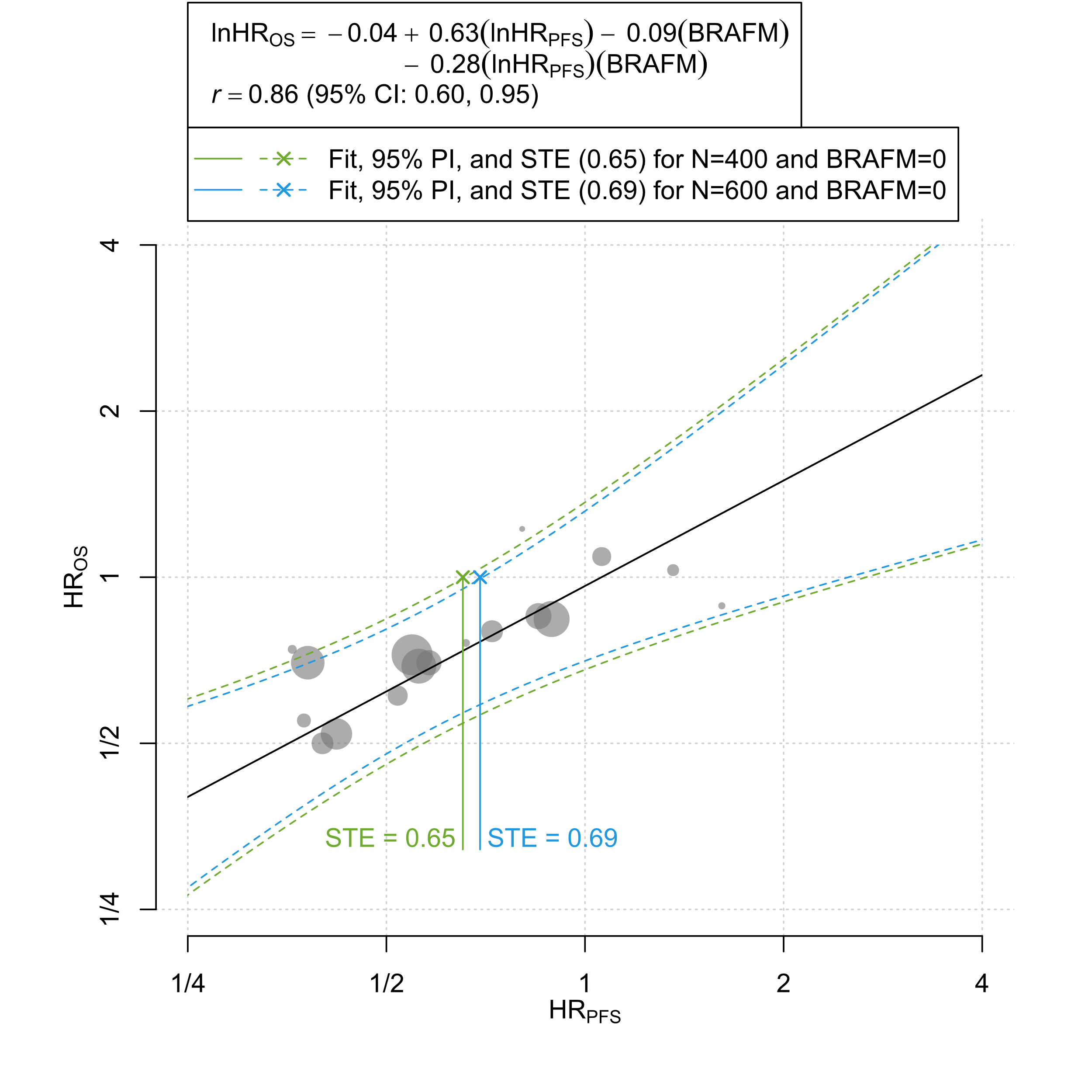


The predictive surrogacy equation (the solid straight line in black) from the multiple WLR adjusting for the proportion of BRAF-MT patients. The demonstration is for the populations consisting of entirely BRAF-WT patients. Each of the plotted gray circles represent the (HR_PFS_, HR_OS_) pair from a treatment-control contrast per trial. Sizes of the circles are proportional to the total number of patients within each contrast. The dotted curves refer to the 95% PIs for the HR_OS_ for a range of HR_RFS_ for hypothetical trials with sample sizes 400 and 600. Solid lines connecting the crosses to the x-axis indicate the STEs calculated for two hypothetical trials with sample sizes 400 (green) and 600 patients (blue). In statistical terms, it corresponds to the HR_PFS_ at which the upper bound of the 95% PI of the HR_OS_ crosses 1. Both axes are on the logarithmic scale.

Abbreviations: CI – Confidence Interval, HR – Hazard Ratio, OS – Overall Survival, PFS – Progression-Free Survival, PI – Prediction Interval, STE – Surrogate Threshold Effect.

**Figure S 3**: Leave-one-out cross validation for the sensitivity analysis including BRAF mutation status as a covariate.


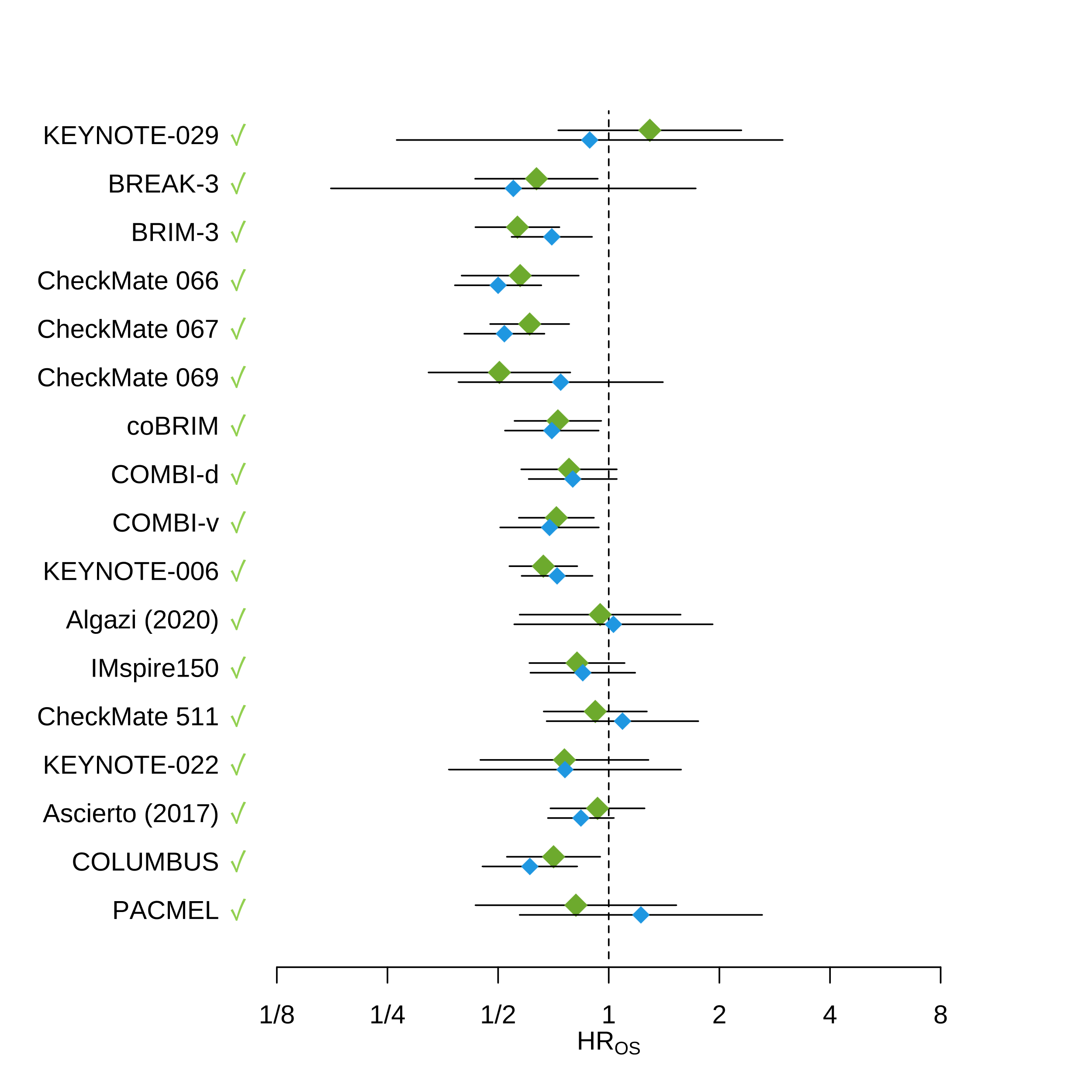


In the figure, the blue diamonds and their error bars represent the HR_OS_’s and their 95% CIs reported from the trials or calculated from reconstructed survival data, respectively. The green diamonds and their error bars represent the predicted HR_OS_’s and their 95% PIs obtained from the WLR, respectively. The green checkmarks and red crosses indicate whether the observed HR_OS_’s were covered by the 95% PIs generated for the HR_OS_’s from the WLR. The x-axis is on the logarithmic scale.

Abbreviations: HR – Hazard Ratio, OS – Overall Survival.

**Figure S 4**: Weighted linear regression plot for the sensitivity analysis including only trials investigating ICIs or BRAF/MEKi.

*
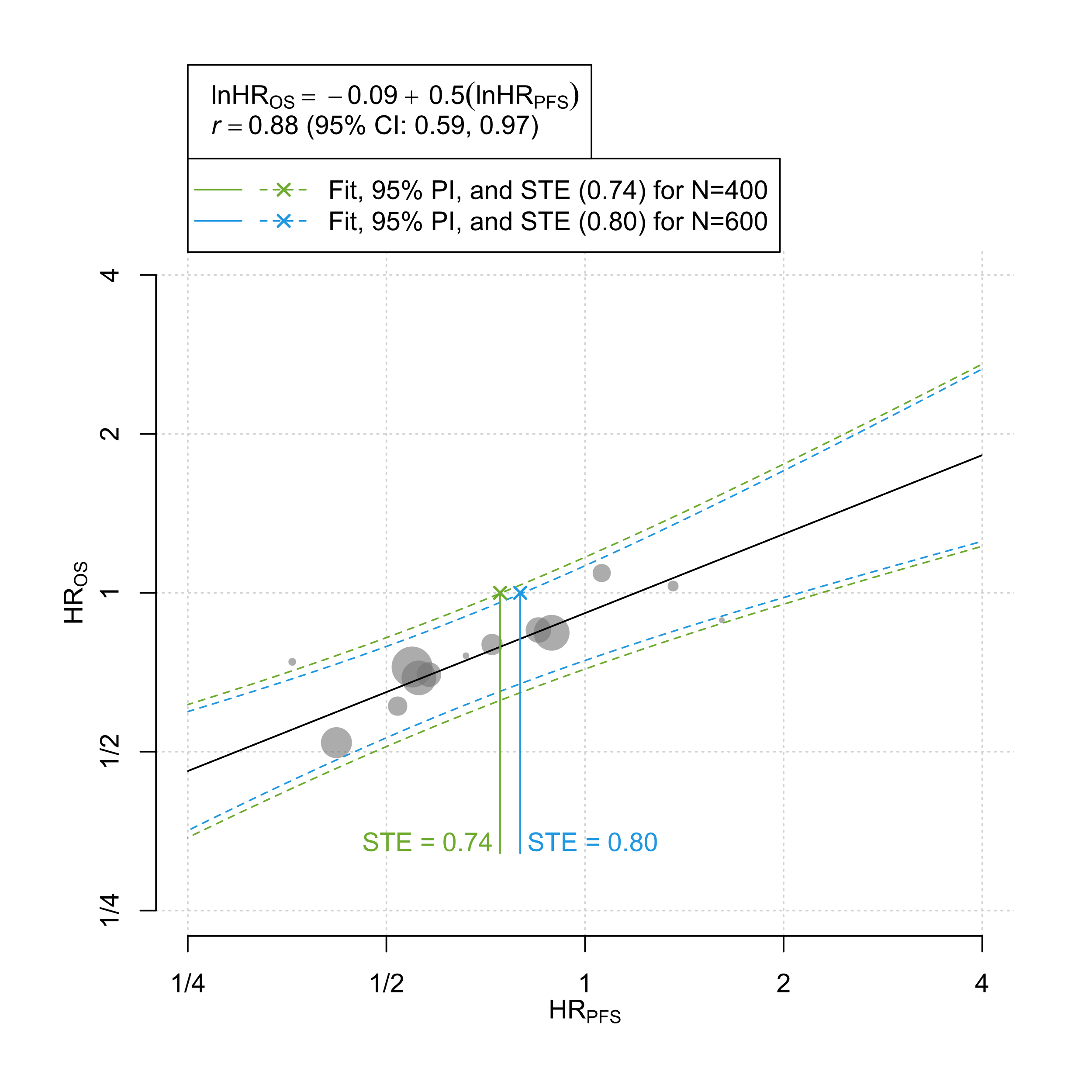
*

The predictive surrogacy equation of ICI- and BRAF/MEKi-only trials is graphed as the solid straight line in black. Each of the plotted gray circles represent the (HR_PFS_, HR_OS_) pair from a treatment-control contrast per trial. Sizes of the circles are proportional to the total number of patients within each contrast. The dotted curves refer to the 95% PIs for the HR_OS_ for a range of HR_RFS_ for hypothetical trials with sample sizes 400 and 600. Solid lines connecting the crosses to the x-axis indicate the STEs calculated for two hypothetical trials with sample sizes 400 (green) and 600 patients (blue). In statistical terms, it corresponds to the HR_PFS_ at which the upper bound of the 95% PI of the HR_OS_ crosses 1. Both axes are on the logarithmic scale.

Abbreviations: BRAF/MEKi – BRAF or mitogen-activated protein kinase inhibitors, CI – Confidence Interval, HR – Hazard Ratio, ICI – Immune Checkpoint Inhibitor, N – Sample size, OS – Overall Survival, PI – Prediction Interval, PFS – Progression-Free Survival, STE – Surrogate Threshold Effect.

**Figure S 5**: Leave-one-out cross validation for the sensitivity analysis including only trials investigating ICIs or BRAF/MEKi.


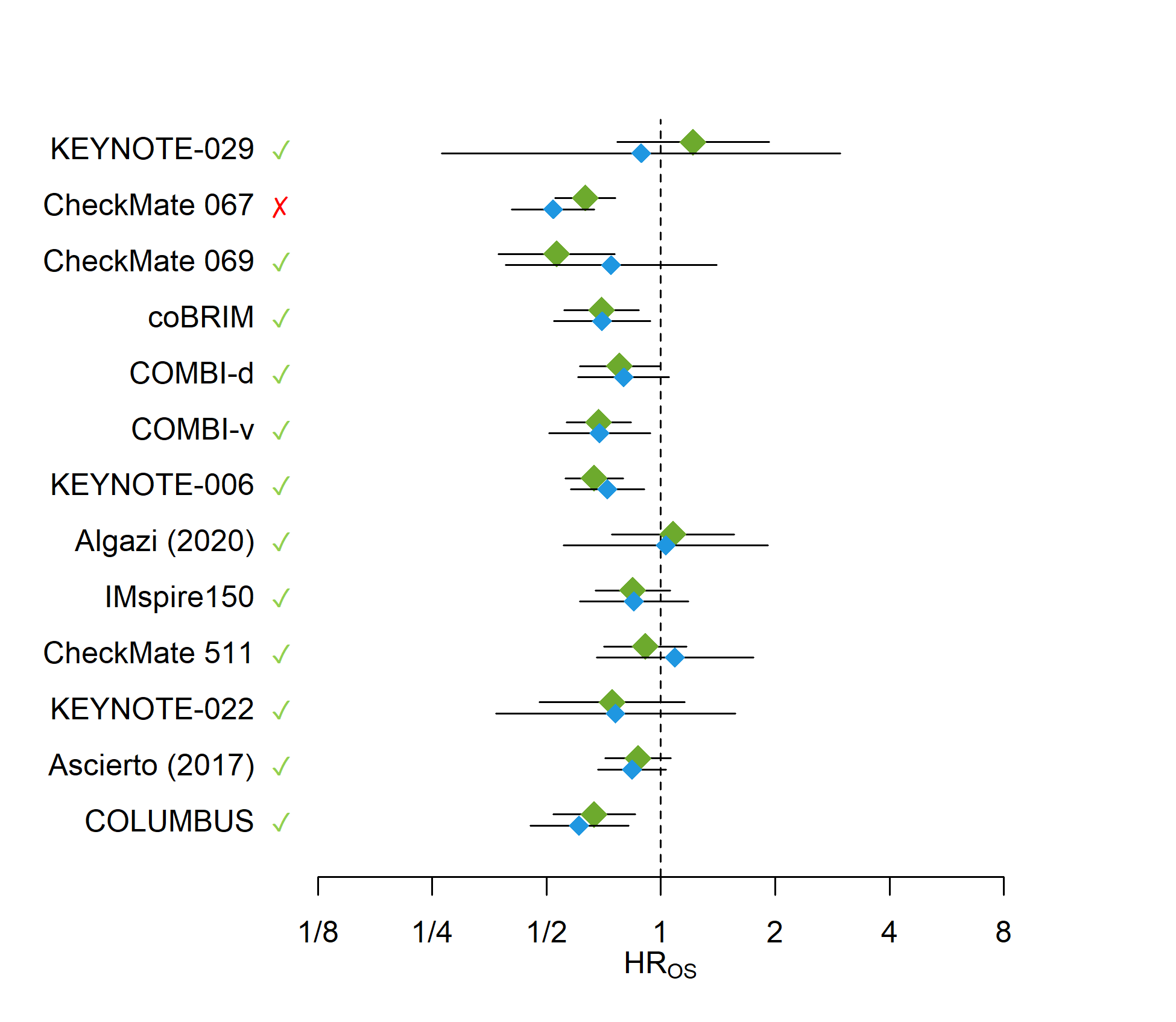


In the figure, the blue diamonds and their error bars represent the HR_OS_’s and their 95% CIs reported from the trials or calculated from reconstructed survival data, respectively. The green diamonds and their error bars represent the predicted HR_OS_’s and their 95% PIs obtained from the WLR, respectively. The green checkmarks and red crosses indicate whether the observed HR_OS_’s were covered by the 95% PIs generated for the HR_OS_’s from the WLR. The x-axis is on the logarithmic scale.

Abbreviations: HR – Hazard Ratio, OS – Overall Survival.

**Figure S 6**: Weighted linear regression plot for the sensitivity analysis including only phase III trials.

*
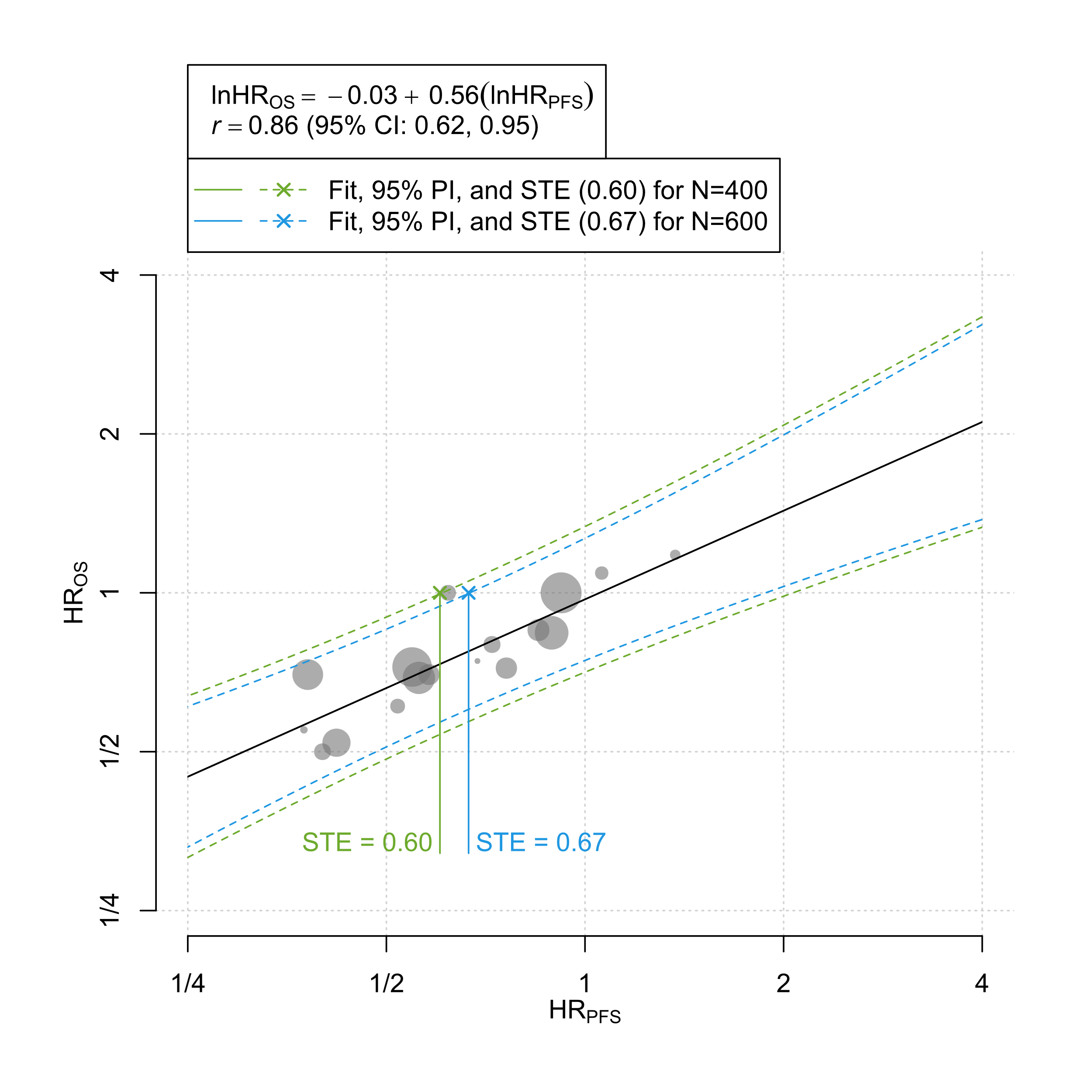
*

The predictive surrogacy equation of phase III only trials is graphed as the solid straight line in black. Each of the plotted gray circles represent the (HR_PFS_, HR_OS_) pair from a treatment-control contrast per trial. Sizes of the circles are proportional to the total number of patients within each contrast. The dotted curves refer to the 95% PIs for the HR_OS_ for a range of HR_RFS_ for hypothetical trials with sample sizes 400 and 600. Solid lines connecting the crosses to the x-axis indicate the STEs calculated for two hypothetical trials with sample sizes 400 (green) and 600 patients (blue). In statistical terms, it corresponds to the HR_PFS_ at which the upper bound of the 95% PI of the HR_OS_ crosses 1. Both axes are on the logarithmic scale.

Abbreviations: CI – Confidence Interval, HR – Hazard Ratio, N – Sample size, OS – Overall Survival, PI – Prediction Interval, PFS – Progression-Free Survival, STE – Surrogate Threshold Effect.

**Figure S 7**: Leave-one-out cross validation for the sensitivity analysis including only phase III trials.


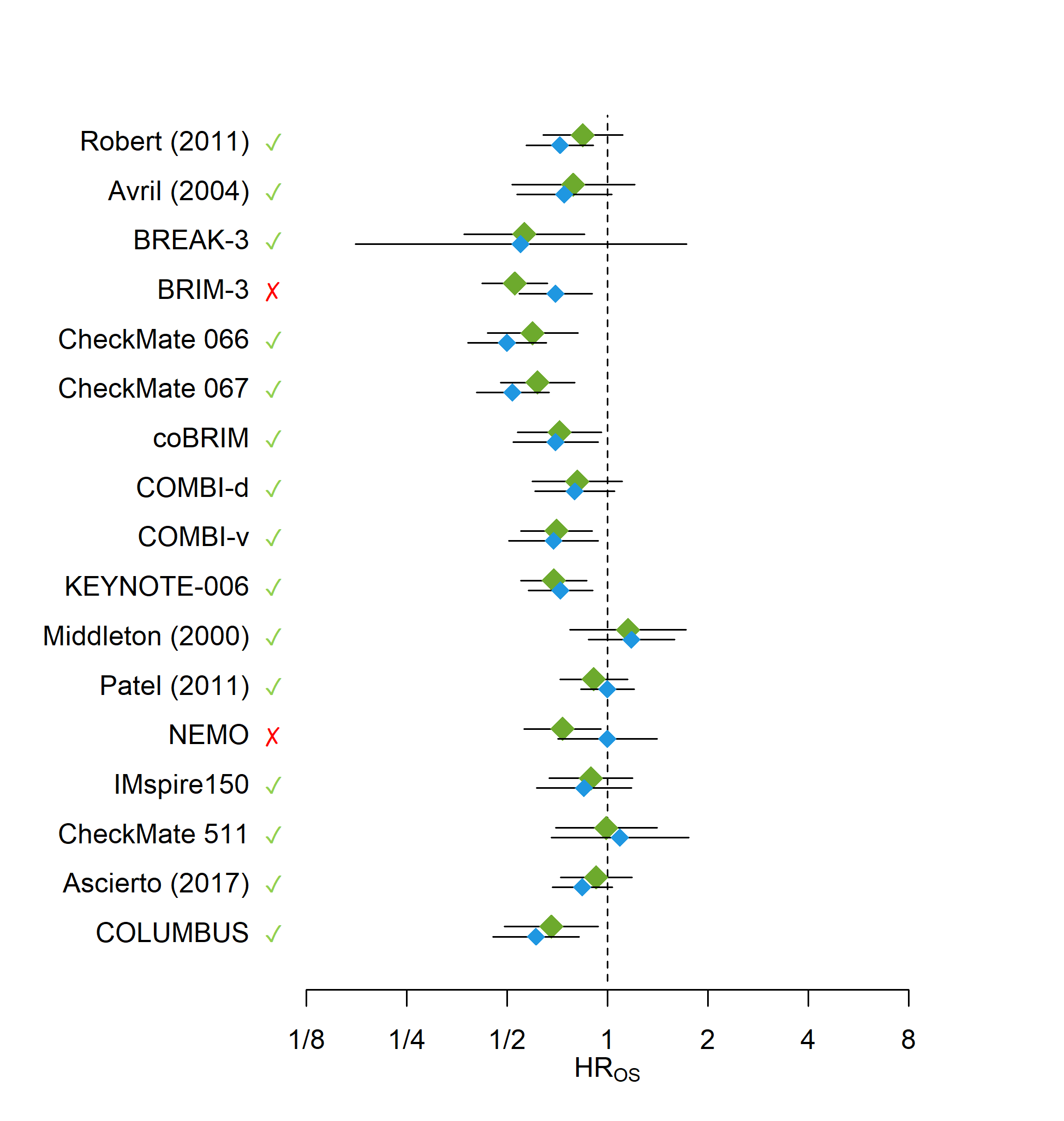


In the figure, the blue diamonds and their error bars represent the HR_OS_’s and their 95% CIs reported from the trials or calculated from reconstructed survival data, respectively. The green diamonds and their error bars represent the predicted HR_OS_’s and their 95% PIs obtained from the WLR, respectively. The green checkmarks and red crosses indicate whether the observed HR_OS_’s were covered by the 95% PIs generated for the HR_OS_’s from the WLR. The x-axis is on the logarithmic scale.

Abbreviations: HR – Hazard Ratio, OS – Overall Survival.

**Figure S 8**: Weighted linear regression plot for the sensitivity analysis including only trials adjusting for crossover.

*
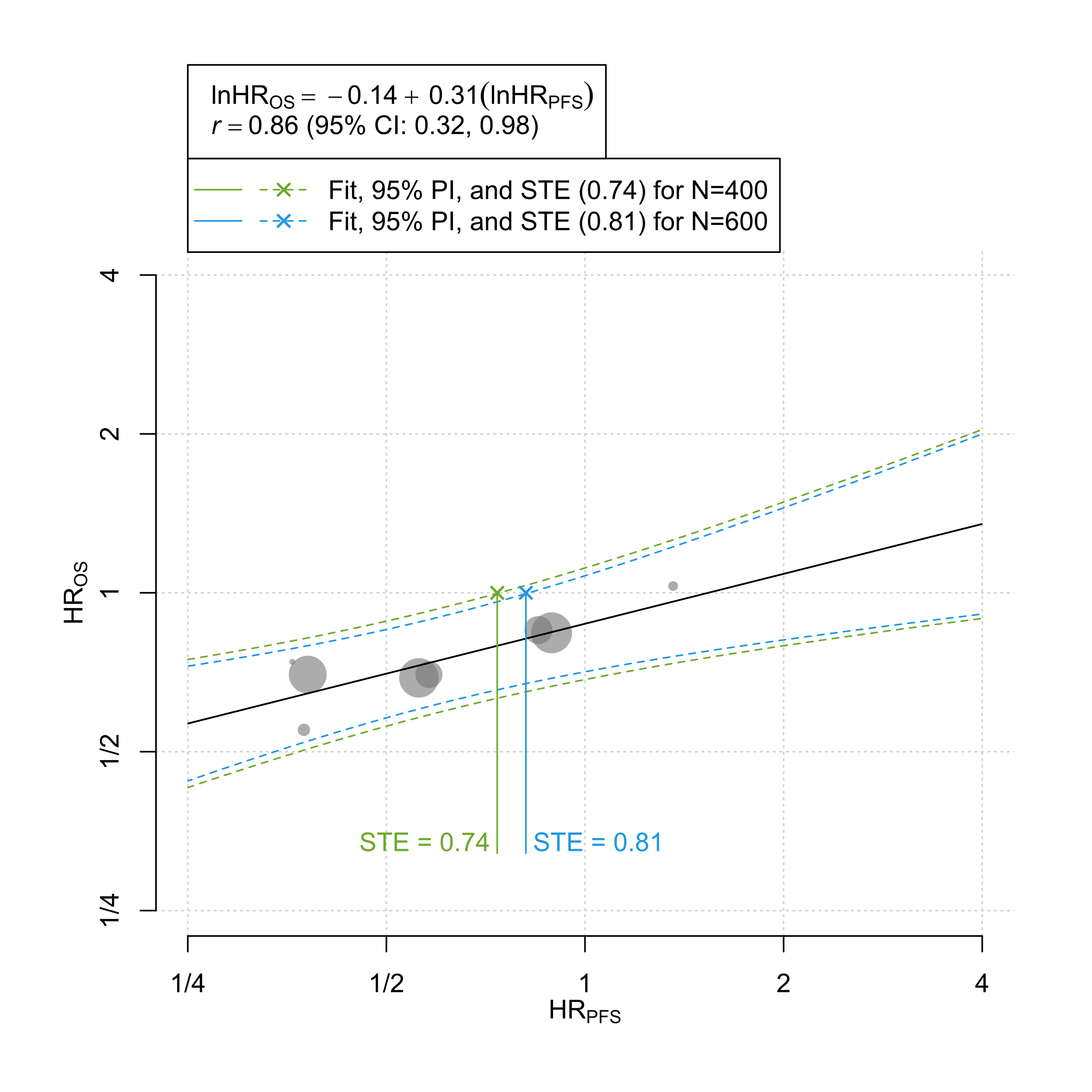
*

The predictive surrogacy equation of the Crossover I analysis included studies that prohibited or adjusted for crossover. The equation is graphed as the solid straight line in black. Each of the plotted gray circles represent the (HR_PFS_, HR_OS_) pair from a treatment-control contrast per trial. Sizes of the circles are proportional to the total number of patients within each contrast. The dotted curves refer to the 95% PIs for the HR_OS_ for a range of HR_RFS_ for hypothetical trials with sample sizes 400 and 600. Solid lines connecting the crosses to the x-axis indicate the STEs calculated for two hypothetical trials with sample sizes 400 (green) and 600 patients (blue). In statistical terms, it corresponds to the HR_PFS_ at which the upper bound of the 95% PI of the HR_OS_ crosses 1. Both axes are on the logarithmic scale.

Abbreviations: CI – Confidence Interval, HR – Hazard Ratio, N – Sample size, OS – Overall Survival, PI – Prediction Interval, PFS – Progression-Free Survival, STE – Surrogate Threshold Effect.

**Figure S 9**: Leave-one-out cross validation for the sensitivity analysis including only trials adjusting for crossover.


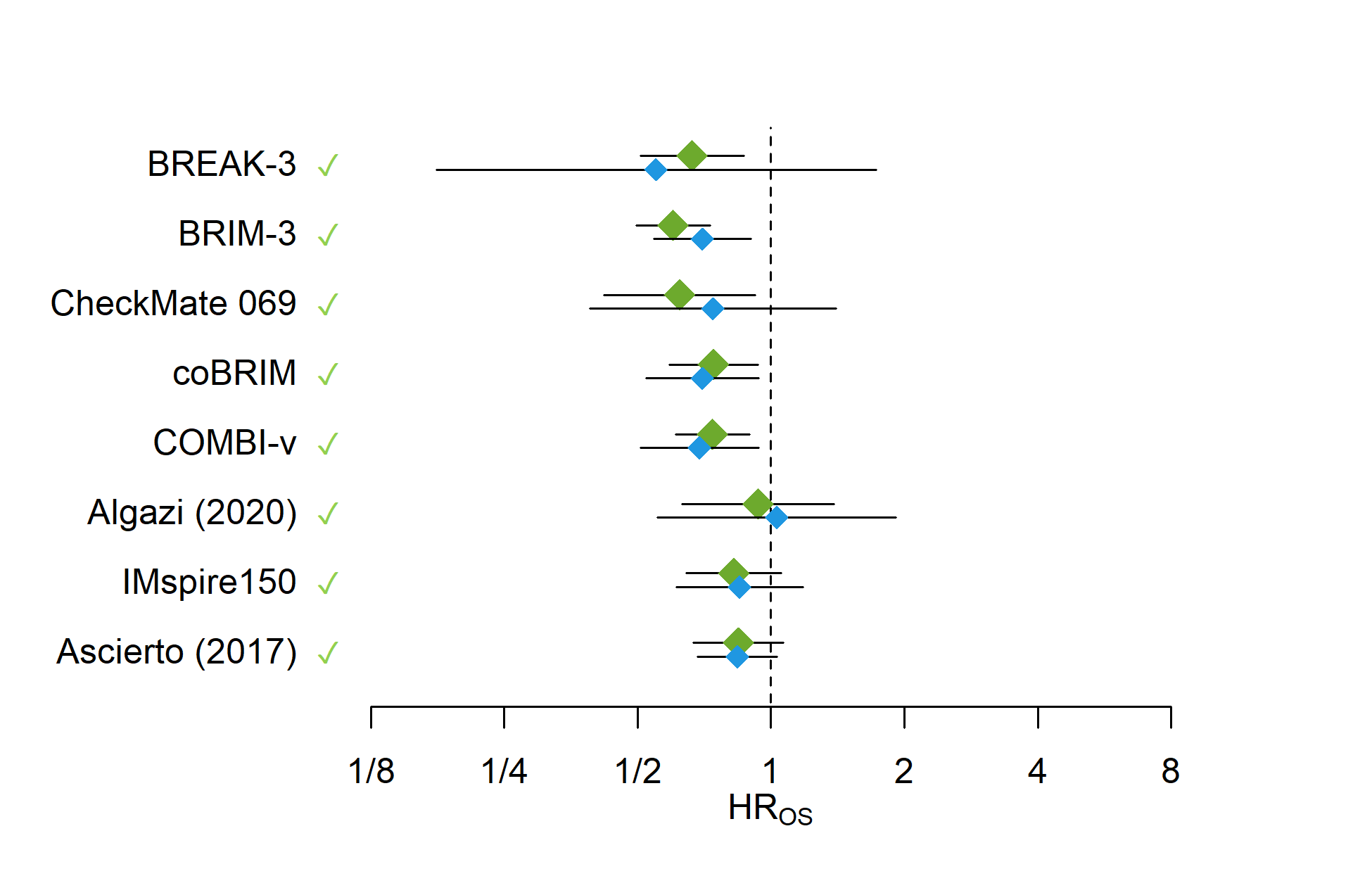


In the figure, the blue diamonds and their error bars represent the HR_OS_’s and their 95% CIs reported from the trials or calculated from reconstructed survival data, respectively. The green diamonds and their error bars represent the predicted HR_OS_’s and their 95% PIs obtained from the WLR, respectively. The green checkmarks and red crosses indicate whether the observed HR_OS_’s were covered by the 95% PIs generated for the HR_OS_’s from the WLR. The x-axis is on the logarithmic scale.

Abbreviations: HR – Hazard Ratio, OS – Overall Survival.

**Figure S 10**: Weighted linear regression plot for the sensitivity analysis including trials that did not violate the proportional hazards assumption.


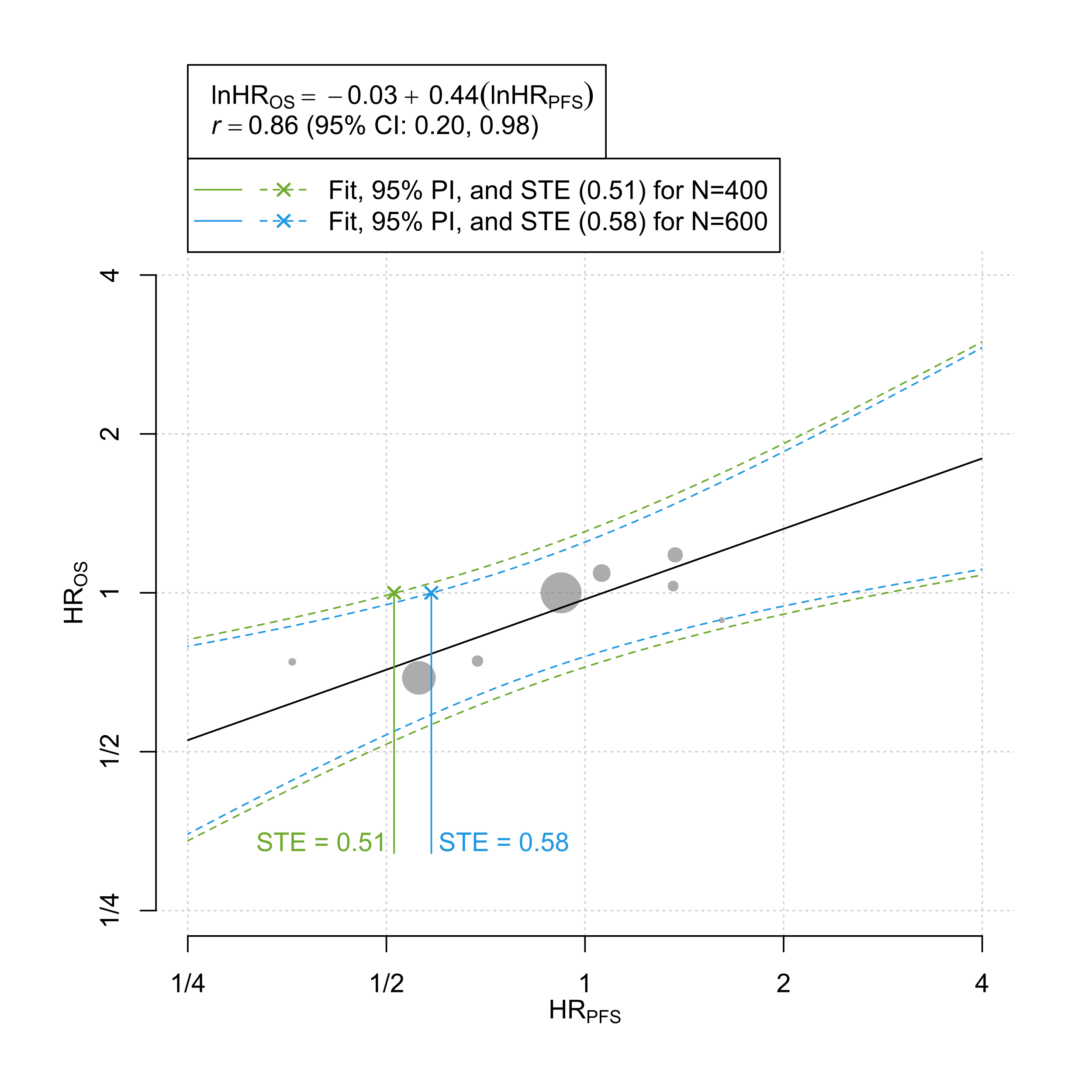


The predictive surrogacy equation for the sensitivity analysis of trials that did not fail the proportional hazards test is graphed as the solid straight line in black. Each of the plotted gray circles represent the (HR_PFS_, HR_OS_) pair from a treatment-control contrast per trial. Sizes of the circles are proportional to the total number of patients within each contrast. The dotted curves refer to the 95% PIs for the HR_OS_ for a range of HR_RFS_ for hypothetical trials with sample sizes 400 and 600. Solid lines connecting the crosses to the x-axis indicate the STEs calculated for two hypothetical trials with sample sizes 400 (green) and 600 patients (blue). In statistical terms, it corresponds to the HR_PFS_ at which the upper bound of the 95% PI of the HR_OS_ crosses 1. Both axes are on the logarithmic scale.

Abbreviations: CI – Confidence Interval, HR – Hazard Ratio, N – Sample size, OS – Overall Survival, PI – Prediction Interval, PFS – Progression-Free Survival, STE – Surrogate Threshold Effect.

**Figure S 11**: Leave-one-out cross validation for the sensitivity analysis including trials that did not violate the proportional hazards assumption.


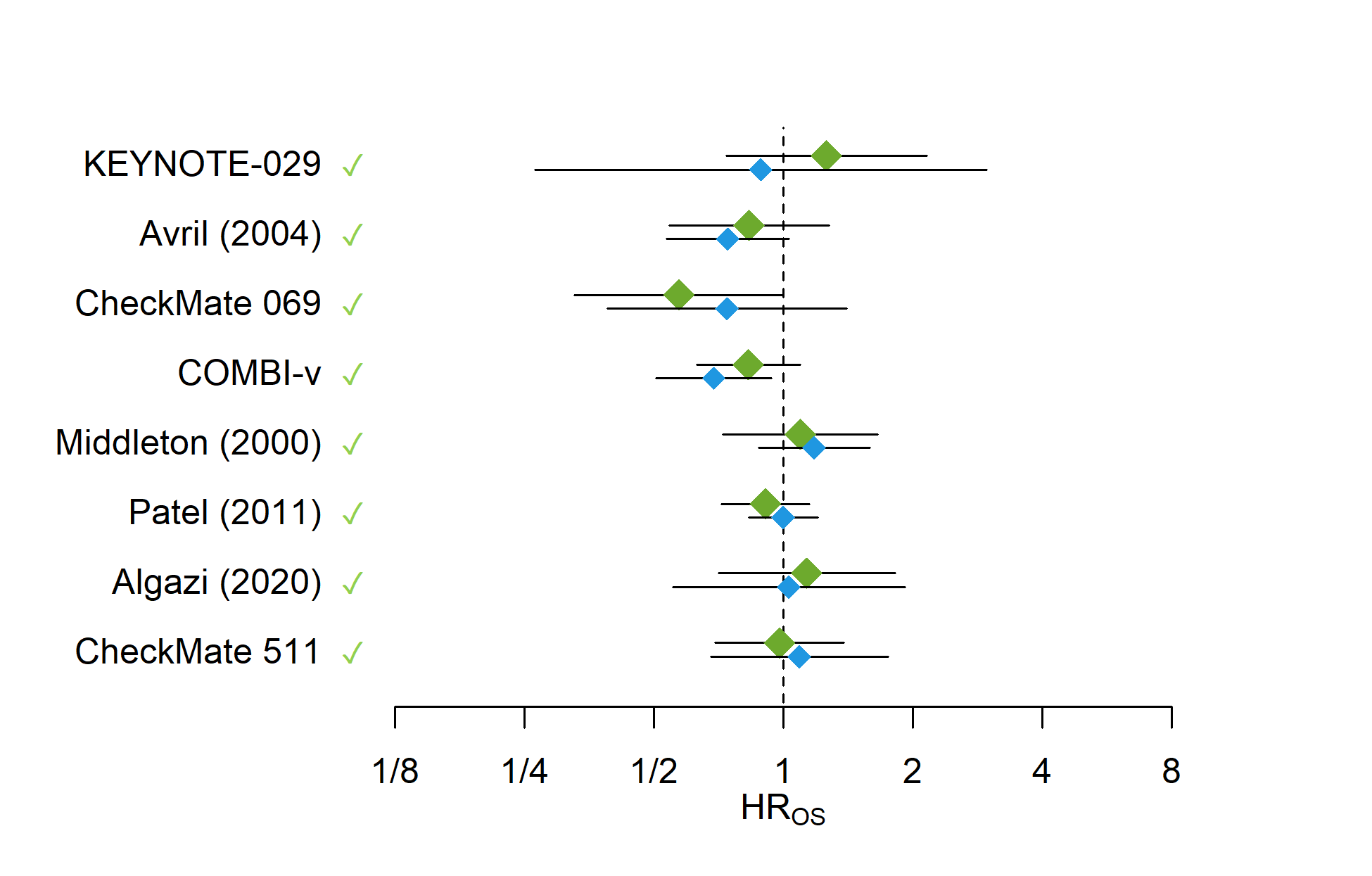


In the figure, the blue diamonds and their error bars represent the HR_OS_’s and their 95% CIs reported from the trials or calculated from reconstructed survival data, respectively. The green diamonds and their error bars represent the predicted HR_OS_’s and their 95% PIs obtained from the WLR, respectively. The green checkmarks and red crosses indicate whether the observed HR_OS_’s were covered by the 95% PIs generated for the HR_OS_’s from the WLR. The x-axis is on the logarithmic scale.

Abbreviations: HR – Hazard Ratio, OS – Overall Survival.

**Figure S 12**: R code for predicting HR_OS_ from HR_PFS_ using the primary model.

predict.OS <- function (PFS_new, N_new, level = 0.95) {

# PFS_new = a numeric vector; the HR of PFS for one or more new trials

# N_new = an numeric vector; the sample size for one or more new trials

# level = a proportion; the desired width of the prediction interval

#

# Example #1: predict.OS(1.15, 446, 0.95)

# Example #2: predict.OS(c(1.15, 1.09, 0.75), c(446, 783, 714), 0.95)

df <- 22

b <- c(-0.0517413367933719, 0.501234715590564)

MSE <- 7.541774

x_new <- log(PFS_new)

V <- matrix(c(0.00186739776444384, 0.00251283364678548,

0.00251283364678548, 0.00583822184893176), nrow=2)

# Calculate prediction variance

Xp <- cbind(1, x_new)

yhat_new_var <- rowSums((Xp %*% V) * Xp)

yhat_pred.var <- yhat_new_var + MSE / N_new

# Calculated yhat with level% PI

yhat_new <- c(Xp %*% b)

quantile <- level + (1 - level) / 2

PI_lwr <- yhat_new - qt(quantile, df) * sqrt(yhat_pred.var)

PI_upr <- yhat_new + qt(quantile, df) * sqrt(yhat_pred.var)

predictions <- data.frame(yhat_new, PI_lwr, PI_upr)

colnames(predictions) <- c("Predicted OS",

sprintf("Lower %s%% PI", level * 100),

sprintf("Upper %s%% PI", level * 100))

# Return predictions with the observed PFS and sample size

cbind.data.frame("Observed PFS" = PFS_new, N = N_new, exp(predictions))

}

**Figure S 13**: Weighted linear regression model, highlighting crossover adjusted studies.


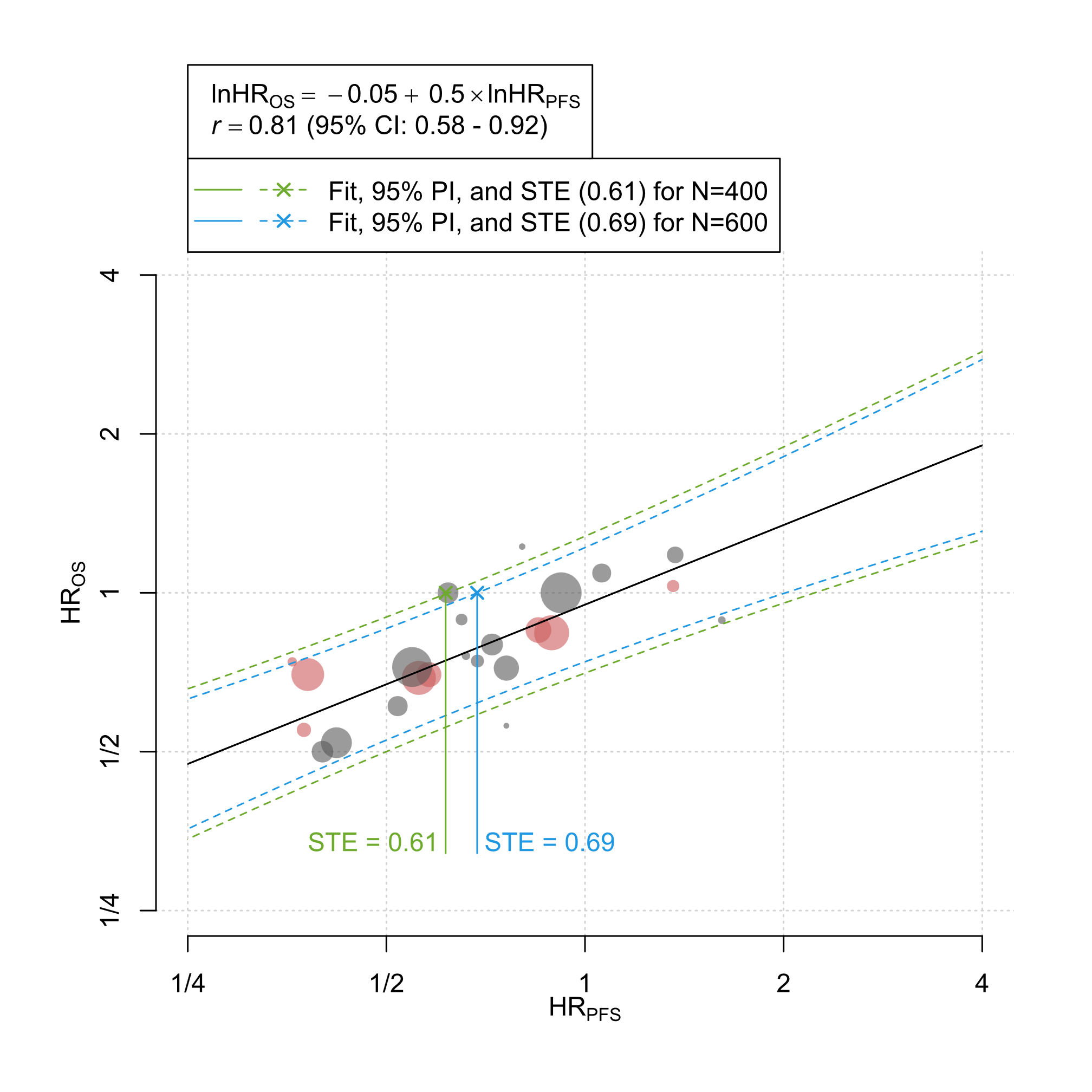


The predictive surrogacy equation is graphed as the solid straight line in black. Each of the plotted circles represent the (HR_PFS_, HR_OS_) pair from a treatment-control contrast per trial; gray circles belong to crossover unadjusted studies, and red circles belong to crossover adjusted studies. Sizes of the circles are proportional to the total number of patients within each contrast. The dotted curves refer to the 95% PIs for the HR_OS_ for a range of HR_RFS_ for hypothetical trials with sample sizes 400 and 600. Solid lines connecting the crosses to the x-axis indicate the STEs calculated for two hypothetical trials with sample sizes 400 (green) and 600 patients (blue). In statistical terms, it corresponds to the HR_PFS_ at which the upper bound of the 95% PI of the HR_OS_ crosses 1. Both axes are on the logarithmic scale.

Abbreviations: CI – Confidence Interval, HR – Hazard Ratio, OS – Overall Survival, PFS – Progression-Free Survival, PI – Prediction Interval, STE – Surrogate Threshold Effect.
